# Central origin of fatigability in Myalgic encephalomyelitis/chronic fatigue syndrome revealed by multimodal neuroimaging

**DOI:** 10.1016/j.nicl.2026.104041

**Published:** 2026-07-28

**Authors:** Patrick Bedard, Kristine M. Knutson, Patrick M. McGurrin, Felipe Vial, Traian Popa, Silvina G. Horovitz, Mark Hallett, Avindra Nath, Brian Walitt

**Affiliations:** a Human Motor Control Section, National Institute of Neurological Disorders and Stroke, National Institutes of Health, Bethesda, MD 20892-1428, USA; b Behavioral Neurology Unit, National Institute of Neurological Disorders and Stroke, National Institutes of Health, Bethesda, MD 20892-1428, USA; c Clínica Alemana Universidad del Desarrollo, Santiago, Plaza Avenue, 680 Las Condes, Chile

**Keywords:** Myalgic encephalomyelitis/chronic fatigue syndrome, Fatigability, fMRI, EEG, EMG, Corticomuscular coherence

## Abstract

Myalgic Encephalomyelitis/Chronic Fatigue Syndrome (ME/CFS) is a debilitating chronic disease characterized by physical and mental fatigue, post-exertional malaise, muscle pain, headaches, and unrefreshing sleep. Fatigability refers to a reduction of muscular force over time despite willed effort and has a central and a peripheral component. We studied whether fatigability in ME/CFS is related to central or peripheral mechanisms.

We recruited fifteen patients with ME/CFS and nineteen age- and sex-matched healthy volunteers (with seven females in each group). Participants performed a fatiguing grip force task, requiring them to maintain 50% of their maximum voluntary force during alternating 30 s blocks of grip and rest. We simultaneously recorded grip force, forearm muscle activity with electromyography, and brain activity with electroencephalography and functional magnetic resonance imaging. Fatigue onset was based on grip force performance and was set individually for each participant.

ME/CFS patients generated the same level of maximum voluntary force than healthy volunteers but developed fatigue much earlier. Healthy volunteers increased their muscle and brain activity from the beginning of the task until the onset of fatigue. Specifically, muscle activity shifted from high to low frequencies and brain activity increased steadily in cortical and subcortical areas. Then, muscle and brain activity declined slowly. In contrast, in ME/CFS, the muscles and brain activity only showed minimal fluctuations across all the task blocks.

The earlier onset of fatigue in ME/CFS is related to central mechanisms, as their brain did not increase its output to drive muscle activity like healthy volunteers did.

While this is a small sample study, and caution should be taken regarding the generalizability of the results, the earlier onset of fatigue in ME/CFS was observed to be related to central mechanisms. The brain in ME/CFS participants did not increase its output to drive muscle activity like healthy volunteers did.

## Highlights

• We studied physical fatigue in patients with Myalgic Encephalomyelitis/Chronic Fatigue Syndrome (ME/CFS) and healthy volunteers with multimodal neuroimaging.
• Participants performed a fatiguing grip force task in alternating grip and rest blocks.
• We simultaneously recorded grip force, brain activity with functional magnetic resonance imaging and electroencephalography, and muscle activity with electromyography.
• ME/CFS fatigued earlier than healthy volunteers. While healthy volunteers increased their brain and muscle activity, ME/CFS only showed minimal fluctuations across all the task blocks.
• We concluded that physical fatigue in ME/CFS is of central nature.

## 1 Introduction

Myalgic Encephalomyelitis/Chronic Fatigue Syndrome (ME/CFS) is a debilitating chronic disorder that affects an estimated 0.9% of the population world-wide (Lim et al., 2020) with no cure or approved treatment. It is characterized by physical and mental fatigue, cognitive fog, post-exertional malaise, muscle pain, and unrefreshing sleep, which causes significant decline in quality of life.

Physical fatigue and mental fatigue are core aspects of the ME/CFS pathophysiology. Fatigability is a reduction of the output of the neuromuscular system that causes a failure to generate enough force to elicit movement despite willed effort and thus refers to an objective decline of performance over time (Enoka and Duchateau, 2016; Kluger et al., 2013). Fatigue refers to the feeling or perception of fatigability (Kluger et al., 2013) and can be independent from fatigability. Fatigability occurs in the brain and in the muscles and can thus have a central or a peripheral origin (Gandevia, 2001).

Very little is known about how the neuromuscular system of patients with ME/CFS adapts during the execution of a motor task that causes fatigability, as few studies have simultaneously measured muscles and whole brain activity (see Siemionow et al., 2004).

We have recently developed methods to address this lack of knowledge in how the neuromuscular system responds during fatigability in ME/CFS, but also in healthy volunteers (HV). We used a grip force task to induce fatigability and simultaneously recorded muscle activity with electromyography (EMG) and brain activity with electroencephalography (EEG) and functional Magnetic Resonance Imaging (fMRI; via the blood oxygen-level-dependent technique: BOLD) (Bedard et al., 2025; Walitt et al., 2024). We showed that fatigability in HV is accompanied by a shift in EMG power from high to low frequencies, an increase in EEG power over the sensorimotor areas, an increase in BOLD signal in the cerebellum and sensorimotor cortical areas, and an increase in corticomuscular coherence (CMC) (Bedard et al., 2025). Other studies in HV reported similar results with EMG (Bigland-Ritchie et al., 1981; Dimitrov et al., 2006; Dimitrova et al., 2005; Zhao et al., 2022b), EEG and fMRI in multiple brain areas often with a delayed ipsilateral contribution (Berchicci et al., 2013; Enders et al., 2016; Guo et al., 2014; Herath et al., 2017; Johnston et al., 2001; Liu et al., 2007; Liu et al., 2003; Schillings et al., 2006; Suviseshamuthu et al., 2022; Wang et al., 2017). CMC changes during fatigability remain controversial, as some studies reported increases (Hsu et al., 2023; Ushiyama et al., 2011) and others decreases (Siemionow et al., 2010; Yang et al., 2009). In ME/CFS, compared to HV (Walitt et al., 2024), we found that people with ME/CFS fatigued earlier during the grip force task, did not show a shift in EMG from high to low frequencies, and showed reduced BOLD signal in the temporal-parietal junction (TPJ).

Here, we aimed to elucidate how the neuromuscular system responds during fatigability in ME/CFS compared to HV. We used different investigational techniques, analyses, and doubled the number of participants compared to our prior work to delve deeper into fatigability in ME/CFS (Bedard et al., 2025; Walitt et al., 2024). A better understanding of fatigability in ME/CFS could resolve whether it is of central or peripheral origin and help inform treatment development.

## 2 Methods

### 2.1 Participants

We recruited fifteen ME/CFS patients and nineteen healthy volunteers (HV; Table 1 and Supplementary Table 1). All participants were right-handed. All ME/CFS and fourteen HV participants were recruited from the *Post-Infectious Myalgic-Encephalomyelitis/Chronic Fatigue Syndrome project at the NIH* (NCT 02669212). Additionally, five HV participants were recruited from the *Post-infection Convalescence of COVID-19 at the NIH* (NCT 04573062). We added these 5 HV to increase statistical power. Eleven of the ME/CFS are the same participants reported on in Walitt et al. (2024) and all HVs were the same participants as in Bedard et al. (2025). Exclusion criteria included any neurological disorder or psychiatric disorders, including depression and anxiety, and active alcohol or illicit drug use. For the HV cohort, exclusion criteria also included the presence of persistent physical or cognitive fatigue. Some participants were taking medications with central nervous system activity. Short-acting central medications (e.g. benzodiazepines, gabapentin, amphetamines, sedative-hypnotics), were held for a minimum of 24 h prior to being studied. Central acting medications with long half-lives (e.g. selective serotonin reuptake inhibitors, serotonin norepinephrine uptake inhibitors) were continued during the study for nine ME/CFS participants. See Supplementary Table 2 for a list of medication for all participants. All ME/CFS participants met at least one published ME/CFS criterion: all met the 1994 Fukuda Criteria and the 2015 Institute of Medicine Criteria, and 9 further met the 2003 Canadian Consensus Criteria. All participants provided informed consent in accordance with the Institutional Review Board of the National Institutes of Health and the Declaration of Helsinki. All participants privacy rights were respected Trial registration: NCT 02669212: 1/29/2016. https: //clinicaltrials.gov/study/NCT02669212. Trial registration: NCT 04573062: 10/01/2020. https://clinicaltrials.gov/study/NCT04573062.

**Table 1:** Participant demographics summary.

	HV	ME/CFS	Statistics (*p*)
Measures			
Age	41.3 ± 13 (20–61)	39.5 ± 12 (19–55)	0.29
Sex	7F, 12 M	7F, 8 M	0.56
Education	3.42 ± 0.9 (1–4)	2.93 ± 0.8 (2–4)	0.11

Means ± sd. Number in parenthesis indicate the range. F: female, M: male. Education was assigned 0: less than high school, 1: high school, 2: some college, 3: college graduate, 4: advanced degree. See *3.1 Demographics* section for more details.

### 2.2 Experimental tasks

#### 2.2.1 Grip force task

**Fig. 1:**
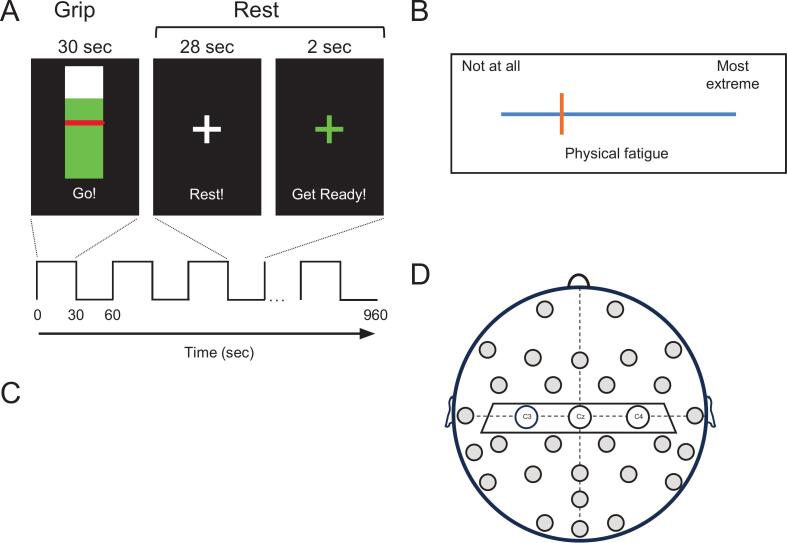
**A**: Task schematic. The grip force task required participants to generate force at 50% of their maximum voluntary contraction (MVC) during 30 s blocks. They used a dynamometer with their dominant right hand. The green portion indicated participant's force level while the static red horizontal bar indicated the 50% MVC. Blocks of 30 s grip were alternating with blocks of 30 s of rest (middle panel). A two *sec* ‘Get Ready!’ signal appeared at the end of each rest block. **B:** Hemodynamic response function (HRF). HRF for the box car fMRI analysis with the five fatigability blocks and the remaining blocks (labelled *Others*). **C:** Visual Analogue Scale (VAS) for the subjective evaluation of mental and physical fatigue before and after the grip force task. **D:** EEG channels. Each circle represents one of the 31 channels recorded. The trapezoid indicates the EEG channels of interest.

We designed an isometric submaximal intermittent grip force task which required participants to maintain 50% of their maximum force during alternating 30 s blocks of grip task and 30 s of rest (Fig. 1A). Participants used their dominant hand to grip an MRI-compatible dynamometer (BIOPAC Systems, Inc., Goleta, CA, USA). Each participant's maximum voluntary contraction (MVC) was determined based on the largest force output recorded from three brief squeezes (less than five seconds) on the dynamometer before entering the scanner.

During the grip task, the level of exerted force was represented by a green bar moving vertically in real time and the target force level (50% of MVC) by a static red horizontal bar (Fig. 1A). The tasks instructions ‘Go!’ was continuously presented during a grip block. During a rest block, tasks instructions’Rest’ and’Get Ready’ were presented for 28 s and 2 s, respectively; the two-second instruction (‘Get Ready’) informed participants of the incoming grip blocks. All visual feedback was presented on a monitor at the foot of the MRI bore via an angled mirror attached to the head coil. We saw the same visual stimuli on a computer in the control room that allowed to monitor task performance.

The paradigm was set for 16 pairs of grip-rest blocks in an fMRI run. If no fatigability developed in the first run, a second fMRI run of 16 blocks was performed after resetting the task (∼1 min); all participants showed signs of fatigability. We kept collecting data even when participant's force level fell below the 50% threshold for several blocks. This gave us flexibility to define offline which blocks to analysis.

#### 2.2.2 Subjective fatigue evaluation

We assessed subjective appraisals of physical and cognitive state fatigue with a visual analog scale immediately before and after the grip force task (∼1 min; Fig. 1B).

### 2.3 Data acquisition

#### 2.3.1 Grip force

We used LabView (National Instruments, LabVIEW) to present the task visual stimuli and record grip force from the dynamometer at 100 Hz. The dynamometer was connected to an amplifier (MP150, BIOPAC Systems Inc.) and a National Instrument multifunction DAQ device for A/D (USB-6212).

#### 2.3.2 Subjective fatigue evaluation

We used SuperLab (Cedrus Corporation, San Pedro, CA) to present visual stimuli and record participant's subjective physical and mental fatigue appraisal. Participants moved a vertical red bar left or right over a visual-analog scale with a hand-held device while in the scanner. We collected subjective fatigue evaluation for thirteen HV and ten ME/CFS, because it was implemented after we had begun the MRI data collection.

#### 2.3.3 Electrophysiology: EMG and EEG

We recorded EMG and EEG with BrainVision Recorder connected to a SyncBox at 5 kHz (Brain Products GmbH, Gilching, Germany). We recorded EMG from the right flexor and extensor carpi radialis muscles (FCR and ECR, respectively) with MRI-compatible electrodes (Ag/AgCl, laminated, carbon composition, 11 mm diameter, part EL508, BioPAC Systems, Inc.) and leads (LEAD108B). We placed the electrodes over the belly of each muscle, identified from palpations and testing visually the EMG response on BrainVision Recorder. We placed the ground electrode over the right elbow.

We used an MRI-compatible 32 channel EEG cap (Brain Products GmbH, Gilching, Germany) with 31 EEG channels and one ECG channel. The scalp of each participant was prepared for EEG recording by cleaning with alcohol and subsequent application of abrasive and conductive gel at each electrode location. ECG was recorded via an electrode placed on participant's left back.

#### 2.3.4 MRI

We acquired MRI data with a 3 T Prisma SIEMENS scanner equipped with a 64-channel head-coil in the Nuclear Magnetic Resonance Center at NIH. First, we collected a high-resolution anatomical sagittal T1-weighted scan with TR = 2200 ms, TE = 4.25 ms, TI = 1000 ms, field of view = 100, FoV: 240 × 240 mm, matrix size: 256 × 256, flip angle = 9°, 160 slices, voxel size 1 mm^3^. Then, we collected T2*-weighted EPI with TR = 2 s, TE = 30 ms, image matrix 64 × 64, flip angle: 70°, FoV: 100, voxel size 3.5 mm^3^. Five participants of the HV group were scanned with a different protocol for the T1-weighted scan with TR = 1900 ms, TE = 3.3 ms, TI = 1100 ms, field of view = 100, FoV: 240 × 240 mm, matrix size: 256 × 256, flip angle = 7°, 176 slices, voxel size 1 mm^3^ and for the T2*-weighted EPI with TR = 2.1 s, multi-echo with 19, 38.21, and 57.42 ms echo times, multi-band acceleration factor of 3, image matrix 84 × 84, flip angle: 60°, FoV: 100, voxel size 2.5 mm^3^.

### 2.4 Data analysis

#### 2.4.1 Grip force

We filtered the grip force with an 8 Hz lowpass Butterworth order of 2 filter. We then normalized grip force to each participant's MVC.

#### 2.4.2 Determination of fatigability onset

We used the grip force data to determine the onset of physical fatigue. First, for each block we calculated the percentage of datapoints above the required threshold of 50% of MVC. We then defined a block as fatigued if less than 90% of datapoints were above that threshold. The onset of physical fatigue was determined to be the first of three consecutive fatigued blocks and was denoted as F_n+1_. The *n* notation indicates that the occurrence of the first fatigued block could differ for each participant, ensuring that fatigability was assessed at the same stage across participants.

To index physical fatigue, we chose five specific blocks during the grip force task. We included the 1st block of the task and the F_n+1_ block. In addition, we included the block preceding the F_n+1_ block, which represented the last successful block before physical fatigue onset and labelled it B_n_, and we included the two blocks following the F_n+1_ block, F_n+2_ and F_n+3_. Thus, the 1st and B_n_ blocks are not continuous, while the B_n_, F_n+1_, F_n+2_, and F_n+3_ blocks are continuous. We did not include blocks in between the 1st and B_n_ blocks because for several ME/CFS participants, their B_n_ block was the second or third block.

#### 2.4.3 Subjective fatigue evaluation

We tabulated participants responses to assess whether fatigue evaluation differed before and after the grip force task and between groups.

#### 2.4.4 Electrophysiology: EMG and EEG

We used EEGLAB, a Matlab toolbox (The MathWorks Inc.), to analyze the EMG and EEG data (Delorme and Makeig, 2004). We first removed the MR gradient-artifacts with the average artifact method (Allen et al., 2000). The process of removing the MR gradient artifact affects the EMG and EEG frequency components, but this method has been shown to be effective in retaining task-related frequencies (Amador-Tejada et al., 2024; Becker et al., 2005; Ritter et al., 2007). Then we used the FMRIB toolbox to identify and remove the ballistocardiogram artifact (Niazy et al., 2005). EMG and EEG data were subsequently downsampled to 1000 Hz.

For EMG, we filtered the data with a high- and then a low-pass filter (20 Hz and 250 Hz respectively) and filtered out line noise of 60 Hz. We did not rectify the EMG. We divided the EMG into 1 s epochs and calculated the Dimitrov Index (DI), an EMG estimate of muscular fatigue (Dimitrov et al., 2006; Dimitrova et al., 2005; Zhao et al., 2022a). DI considers the whole power spectrum in its computation and is thus less biased than a subjectively chosen low/high ratio and is more sensitive than the median or mean frequency (Puce et al., 2021). We also calculated the EMG amplitude with the root-mean-square of the EMG signal (RMS). RMS of EMG indicates the amount of power and intensity of the muscle contraction and relates to the amount of muscles fiber activated.

For EEG, we filtered the data with a high- and then a low-pass filter (0.5 Hz and 55 Hz respectively). We ran Independent Component Analysis (ICA) and use the *iclabel* function to automatically classify components in seven categories including brain, muscle, eye, heart, line noise, channel noise, and other (Pion-Tonachini et al., 2019). We removed the non-brain components if the probability of a component being correctly labelled was at least 70%. We used the *clean_artifacts* in EEGLAB function to remove bad sections and bad channels. We visually inspected the data and components to further remove noisy components and bad sections if necessary. We interpolated the bad channels and re-referenced to the common average of all channels. We repeated the ICA and *iclabel* steps and kept only the brain components that were above 70%. To estimate EEG power, we divided the EEG into 1 s epochs, as done with EMG, and then used the *bandpower* function in Matlab. The function calculates power in frequency bands for the theta, mu, beta, and gamma bands using 4–8, 8–13, 13–30, and 30–50 Hz, respectively. We assessed fatigability-related changes in a subset of the channels related to sensorimotor control, including C3, Cz and C4 (inside the trapezoid in Fig. 1D). We also calculated the spectrogram for each participant (*pspectrum* function in Matlab) within the 0–50 Hz range and a time resolution of 1 s, then smoothed with a kernel of 10 Hz and 6 s and finally averaged across participants for each group.

#### 2.4.5 Corticomuscular coherence directionality

We assessed corticomuscular coherence (CMC) in the efferent (brain to muscles) and afferent (muscles to brain) direction between the EEG electrode C3 and the forearm muscles (ECR and FCR) in the beta and gamma bands. We did not rectify the EMG, in keeping with prior literature (Delcamp et al., 2023; Glories et al., 2021; McClelland et al., 2012; Neto and Christou, 2010). We used the NeuroSpec2.11 toolbox with the *sp2a2_R2* function (Halliday, 2015; Halliday et al., 2016). This provides non-parametric assessment of directional coherence estimates. CMC was calculated by dividing the cross-spectrum between EMG and EEG by the auto-spectrum of EMG and EEG signal in the time-frequency domain. CMC indicates the linear connection between EEG and EMG and is an extension of Pearson correlation coefficient in the frequency domain. This tool builds time segments based on the power of 2 which resulted in 29 segments per 30 s block with a temporal resolution of 1.024 s and a frequency resolution of 0.976 Hz. Thus, for each of the five blocks of 30,000 timepoints, we analyzed the first 29,696.

#### 2.4.6 MRI

We used AFNI (Cox, 1996) to process the anatomical and EPI data with the *afni_proc.py* tool. The EPI of the five participants with a different scanning protocol and a voxel size of 2.5 mm^3^ were resampled to 3.5 mm^3^ to match the other participants. The analysis included despiking the timeseries, registering the EPI data to the anatomical scan, adjusting for slice timing offsets, motion correcting the timeseries with reference to the first collected image with rigid body transformations using cubic polynomial interpolation, and spatially blurred the timeseries with a 6 mm FWHM Gaussian kernel. We applied a band-pass filter 0.01–0.1 Hz, removed CSF signal, and also used @ANATICOR to remove white matter signal from the timeseries (Jo et al., 2010). We set the motion limit at 3 mm and identified volumes with more than 10% of outliers as defined with *3dToutcount* function in AFNI in a censor file to be used subsequently in the regression analysis. The anatomical and the EPI timeseries were transformed to the MNI template with linear transformations.

To reveal fatigability-related brain activation for each participant, we ran multiple regression analysis with the *3dREMLfit* function in AFNI. This function uses generalized linear squares with restricted maximum likelihood estimation of the temporal auto-correlation structure of the timeseries. We formed separate regressors for each of the five blocks of interest (1st, B_n_, F_n+1_, F_n+2_, F_n+3_) by convolving the onset times of each block with a square wave to form a boxcar block design. The remaining blocks were included in a separate regressor labelled *Others* (Fig. 1C). The regression equation is thus:

BOLD = ß_0_ + ß_1_X_1_ + ß_2_X_2_ + ß_3_X_3_ + ß_4_X_4_ + ß_5_X_5_ + ß_others_X_others_ + ε,

where ß_0_ is the intercept, ß_1–5_ are the estimated regressors for each of the five blocks of interest, ß_others_ is the estimated regressor for the *Others* blocks, X_1–5_ are the predictors for each of the five blocks, X_others_ is the predictor for the *Others* blocks, and ε is the error. An example of the HRF model is shown in Fig. 1C. We also included in the model the demeaned and derivatives of head motion parameters and censored out bad volumes identified in the censor file.

### 2.5 Group analysis and statistics

#### 2.5.1 Grip force, EEG, EMG, and CMC

We did not include the first five epochs (i.e. five seconds) of each grip block because force was at zero to start a block and participants had to generate considerable force to reach the 50% of MVC. Due to this instability, these data are unsuitable for our analyses, especially for time-frequency analysis. For CMC, we removed the first four segments to have the same number of epochs as with the EMG and EEG.

Since fatigability could manifest itself within or across the five blocks (1st, B_n_, F_n+1_, F_n+2_, F_n+3_), we assessed fatigability-related changes within and across blocks. To assess within block effects, we used robust regression (*fitlm* function in Matlab) to obtain the beta weights representing the rate of change (i.e., slopes) across the 25 epochs for each participant. To assess across blocks effects, we calculated the average of each of the five blocks (i.e., all 25 epochs) for each participant.

At the group level, the beta weights representing the rate of change and the averages per block were separately analyzed with a two-way ANOVA with Groups (HV, ME/CFS) and Blocks (1st, B_n_, F_n+1_, F_n+2_, F_n+3_) as repeated measures and participants as a random factor. We included age and sex as covariates. We used the function *fitlme* in Matlab which is a linear mixed-effects analysis. Virtues of linear mixed-effects analysis over traditional ANOVA include better handling of unbalanced design such as unequal number of participants per group or missing data points and allows for violation of compound symmetry, amongst other advantages (Chen et al., 2013). We used the Greenhouse-Geisser correction in cases of non-sphericity as measured by the Mauchly test. The threshold for statistical significance was set at a *p*-value of 0.05. We contrasted the two groups at each block separately with a two-sample *t*-test and assessed within group changes between pairs of blocks (e.g., 1st vs F_n+1_) with a paired *t*-test. These multiple comparisons were corrected with the False Discovery Rate (FDR) (Benjamini and Hochberg, 1995) at a *p* ≤ 0.05. We also used robust regression across the five blocks (slopes or averages), depending on the results profile to assess the change in trends across blocks. We removed outliers in each group defined as values that are more than three times the mean absolute deviations from the median. Data in the text and figures are reported as means ± standard error of the means (s.e.m.).

#### 2.5.2 MRI

At the group-level, we assessed fatigability-related activation with the *3dLME* function in AFNI (Chen et al., 2013) to run linear mixed effects two-way ANOVA with Groups (HV, ME/CFS) and Blocks (1st, B_n_, F_n+1_, F_n+2_, F_n+3_) as repeated measures factors and participants as a random factor. We included age, sex, and the two MRI protocols as covariates. We restrained the analysis to the grey matter with the Brainnetome atlas and SUIT cerebellar atlas (Diedrichsen, 2006; Fan et al., 2016). We thresholded the activation brain map with a *p* ≤ 0.05 and corrected for multiple comparisons using a cluster threshold of *p* ≤ 0.05 resulting in clusters having at least 201 contiguous significant voxels; that number comes from using the residuals of the times series from the participant's regression (*3dREMLfit*) with the AutoCorrelation Function to estimate the noise smoothness.

We also implemented an ROI based analysis to complement the previous analysis. We only implemented the ROI analysis in areas of interest in which the whole-brain approach failed to reveal significant effects and in areas in which we found fatigability-related effects in our previous work (Bedard et al., 2025). This ROI analysis differs from the whole-brain analysis as we averaged all the voxels within an ROI and then analyzed with the two-way ANOVA. The whole-brain approach is performed on each voxel separately, and if enough significant voxels form a statistically large enough cluster, it is deemed significant. We used the same two-way ANOVA function *(fitlme* in Matlab) as with the other metrics and we included age, sex, and the two MRI protocols as covariates. We also used a *p* ≤ 0.05 and corrected for multiple comparisons with FDR. We formed ROI (Fig. 6A) in the bilateral primary motor cortex, pre-motor cortex, supplementary motor area, and somatosensory cortex (M1, PM, SMA, SS, respectively) with the Brainnetome atlas (Fan et al., 2016). For the somatosensory cortex, the atlas has four bilateral areas, and we pooled these into one ROI per hemisphere.

## 3 Results

### 3.1 Demographics

Age did not differ significantly between the HV and ME/CFS groups (Table 1; 41.1 ± 3 vs. 39.5 ± 3, *t*(32) = 1.08, *p* = 0.29), nor did the sex distribution in the HV (7/19 F) and ME/CFS (7/15 F) groups (*χ*^*2*^ = 0.33, *p* = 0.56), nor did the education level between the HV and ME/CFS groups (*t*(32) = 1.65, *p* = 0.11).

### 3.2 Behavioral performance

**Fig. 2:**
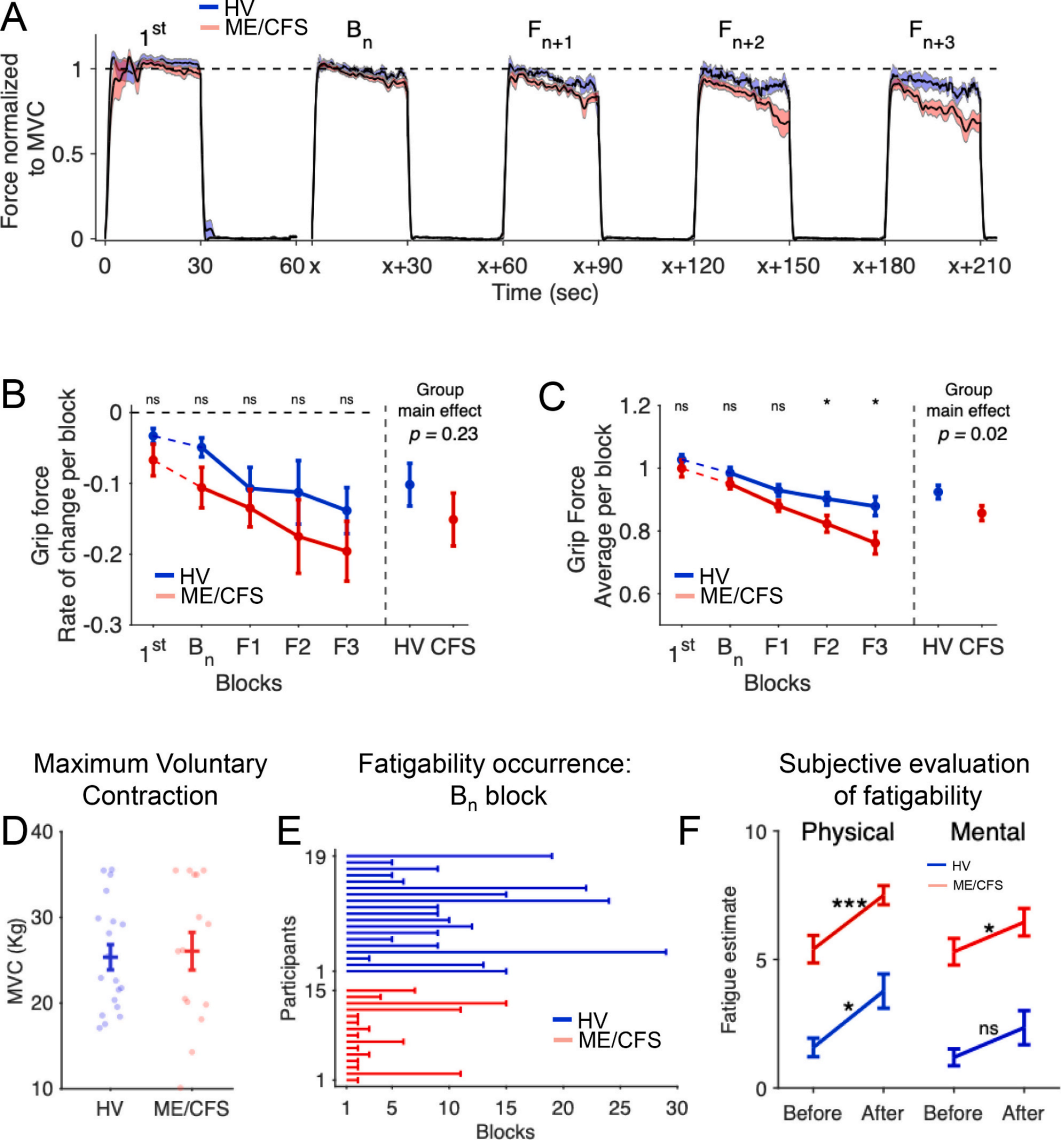
Behavioral performance for the HV and ME/CFS groups. **A:** Grip force normalized to MVC for each of the five blocks. The thick black line and shaded area indicate the group average ± s.e.m., respectively. The horizontal dashed line represents the normalized force level at 50% of MVC. Here, we show the first five seconds to niceify the figure, but for the analysis, the first five seconds were removed. **B:** Grip force rate of change within each block (means ± s.e.m.). F1, F2 and, F3 are the F_n+1_, F_n+2_, and F_n+3_ blocks, respectively. **C:** Grip force averaged per block (means ± s.e.m.). **D:** Maximum Voluntary Contraction (MVC) for each participant and group (means ± s.e.m.). No significant group difference was noted. **E:** Occurrence of the B_n_ block. The ME/CFS fatigued faster and so reached the B_n_ block (i.e., last successful block) earlier than the HV. **F:** Subjective evaluation of mental and physical fatigue before and after the grip force.

Grip force level (Fig. 2A) for the 1st and B_n_ blocks was, as expected, comparable between the ME/CFS and HV. Then, grip force decreased for both groups within and across blocks, but this decline was faster for ME/CFS than HV. For the slopes within blocks (Fig. 2B; F1, F2 and, F3 refer to the F_n+1_, F_n+2_, and F_n+3_ blocks, respectively), the two-way ANOVA did not reveal any significant Groups main effect, or interaction, but there was a significant main effect of Blocks, due to the negative slopes getting steeper across blocks (Groups: F(1, 34) = 1.49, *p* = 0.23; Blocks: F(4, 134) = 6.62, *p* < 0.001; Interaction: F(4, 134) = 0.17, *p* = 0.95). Group contrasts at each block revealed no significant difference (all *p* > 0.32). For the grip force average per block (Fig. 2C), the two-way ANOVA revealed a significant Groups main effect, a significant Blocks main effect, and a significant interaction (Groups: F(1, 34) = 6.18, *p* = 0.02; Blocks: F(4, 134) = 50.67, *p* < 0.001; Interaction: F(4, 134) = 2.83, *p* = 0.03). Group contrasts for each block showed no significant difference at the 1st, B_n_, and F_n+1_ blocks, but ME/CFS had significantly lower grip force than the HV at the F_n+2_ and F_n+3_ blocks (*p* = 0.95, 0.16, 0.085, 0.04, 0.04 across the five blocks). Further, the decline in force across the five blocks was significantly greater for the ME/CFS than the HV (slopes: −6.1 ± 4 vs. -3.8 ± 3, *t*(32) = 2.10, *p* = 0.04). Thus, grip force did not differ between groups for the 1st and B_n_ blocks, i.e. before physical fatigue onset, bur grip force declined faster for the ME/CFS.

MVC did not differ significantly between the groups (Fig. 2D; *t*(32) = 0.27, *p* = 0.78).

The occurrence of the B_n_ block happened significantly later for the HV than the ME/CFS (Fig. 2E; 12.0 ± 2 vs. 4.9 ± 1, *t*(32) = 3.39, *p* = 0.002).

### 3.3 Subjective fatigue evaluation

ME/CFS reported more physical and mental fatigue than the HV before and after the grip task (Fig. 2F; all *p* < 0.001). Both groups reported more physical fatigue after performing the grip task compared to before (ME/CFS: *t*(12) = 2.82, *p* = 0.02; HV: *t*(9) = 3.95, *p* = 0.003). However, only the ME/CFS group showed a significant increase in mental fatigue after the task (ME/CFS: *t*(9) = 2.64, *p* = 0.03; HV: *t*(12) = 1.36, *p* = 0.20). Thus, ME/CFS reported being more fatigued physically and mentally before the task and became even more fatigued after the task.

### 3.4 EMG

**Fig. 3:**
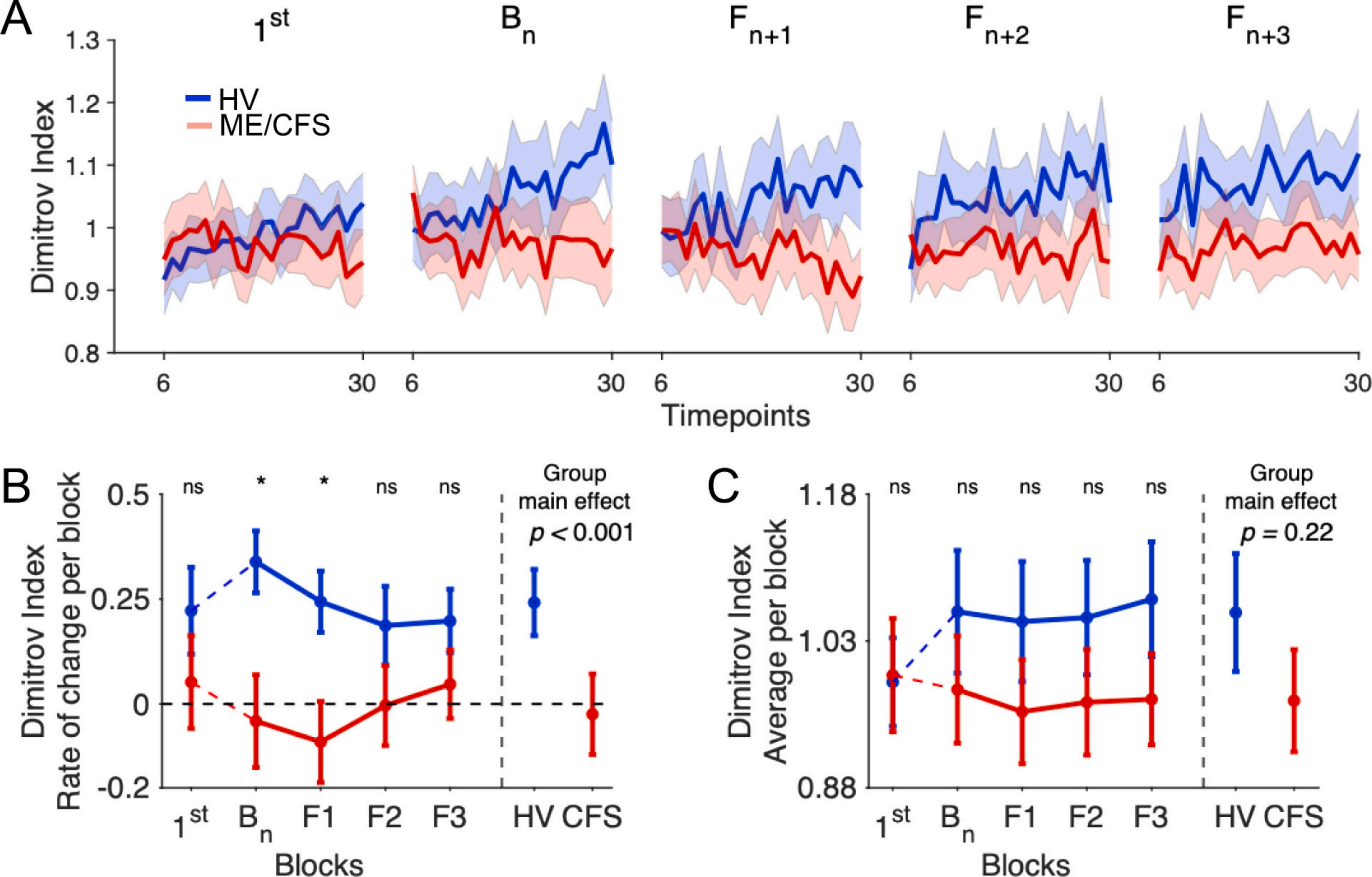
EMG analyzed with the Dimitrov Index (DI). **A:** DI within and across the five task blocks and 25 timepoints for each group (means ± s.e.m.). HV had higher DI values and steeper slopes within each block compared to ME/CFS. **B:** DI rate of change within each block (means ± s.e.m.). The slopes were significantly steeper for HV than ME/CFS. Group differences at each block are indicated by the */**/***/ns at the top of the panel. **C:** DI averages of each of the five blocks. DI increased for the HV but decreased for the ME/CFS across the five blocks. We also provide the *p* value of the Group main effects of the two-way ANOVA, with group means ± s.e.m., to the right of the rate of change within each block (lower-left panel), and averages of each of the five blocks (lower-right panel). The dashed lines between the 1st and B_n_ block is to show that the blocks were not continuous. *: *p* < 0.05, **: *p* < 0.01, ***: *p* < 0.001, *ns*: not significant.

Dimitrov index. HV had higher DI for each block and their DI increased within each of the blocks while for ME/CFS DI remained at the same level throughout the task (Fig. 3A). For the slopes within blocks (Fig. 3B), the two-way ANOVA revealed a significant Groups main effect, and no significant Blocks main effect or significant interaction (Groups: F(1, 34) = 14.31, *p* < 0.001; Blocks: F(4, 134) = 0.28, *p* = 0.89; Interaction: F(4, 134) = 0.79, *p* = 0.53). The significant Groups main effect indicated steeper positive slopes for the HV than ME/CFS Group for all blocks. Group contrasts for each block showed significant difference at block B_n_ and F_n+1_ (*p* = 0.28, 0.02, 0.02, 0.26, 0.26 across the five blocks). Also, for the HV, the slopes were significantly greater than 0 for all blocks but the 1st and F_n+2_ (*p* = 0.057, 0.001, 0.009, 0.062, 0.03, across the five blocks). For the ME/CFS none of the slopes were significantly different than 0 (all *p* > 0.87).

For the DI averages of each block (Fig. 3C), the two-way ANOVA revealed a significant interaction and no significant Groups or Blocks main effect (Groups: F(1, 34) = 1.59, *p* = 0.22; Blocks: F(4, 134) = 0.59, *p* = 0.67: Interaction: F(4, 134) = 3.62, *p* = 0.007). In HV, DI increased significantly from the 1st to the B_n_ block (*t*(18) = 2.17, *p* = 0.04), but for the ME/CFS DI decreased slightly, but not significantly (*t*(14) = 0.42, *p* = 0.68). This opposite direction of DI between the 1st and the B_n_ block gave a group difference that approached statistical significance (*t*(32) = 1.93, *p* = 0.08). Group contrasts for each block showed no significant difference (all *p* > 0.24).

RMS. For the slopes within blocks (Supplementary Fig. 1), the two-way ANOVA revealed a significant Blocks main effect and no significant Groups main effect or significant interaction (Groups: F(1, 34) = 1.07, *p* = 0.31; Blocks: F(4, 134) = 4.22, *p* = 0.003: Interaction: F(4, 134) = 1.51, *p* = 0.20). The Blocks main effect was due to a decrease of the RMS slopes steepness across blocks. For the averages per block there was no significant Groups or Blocks main effect or significant interaction (Groups: F(1, 34) = 0.41, *p* = 0.53; Blocks: F(4, 134) = 1.08, *p* = 0.37: Interaction: F(4, 134) = 0.86, *p* = 0.49).

In summary, DI for the HV was higher across blocks and increased more within blocks compared to ME/CFS.

### 3.5 EEG

**Fig. 4:**
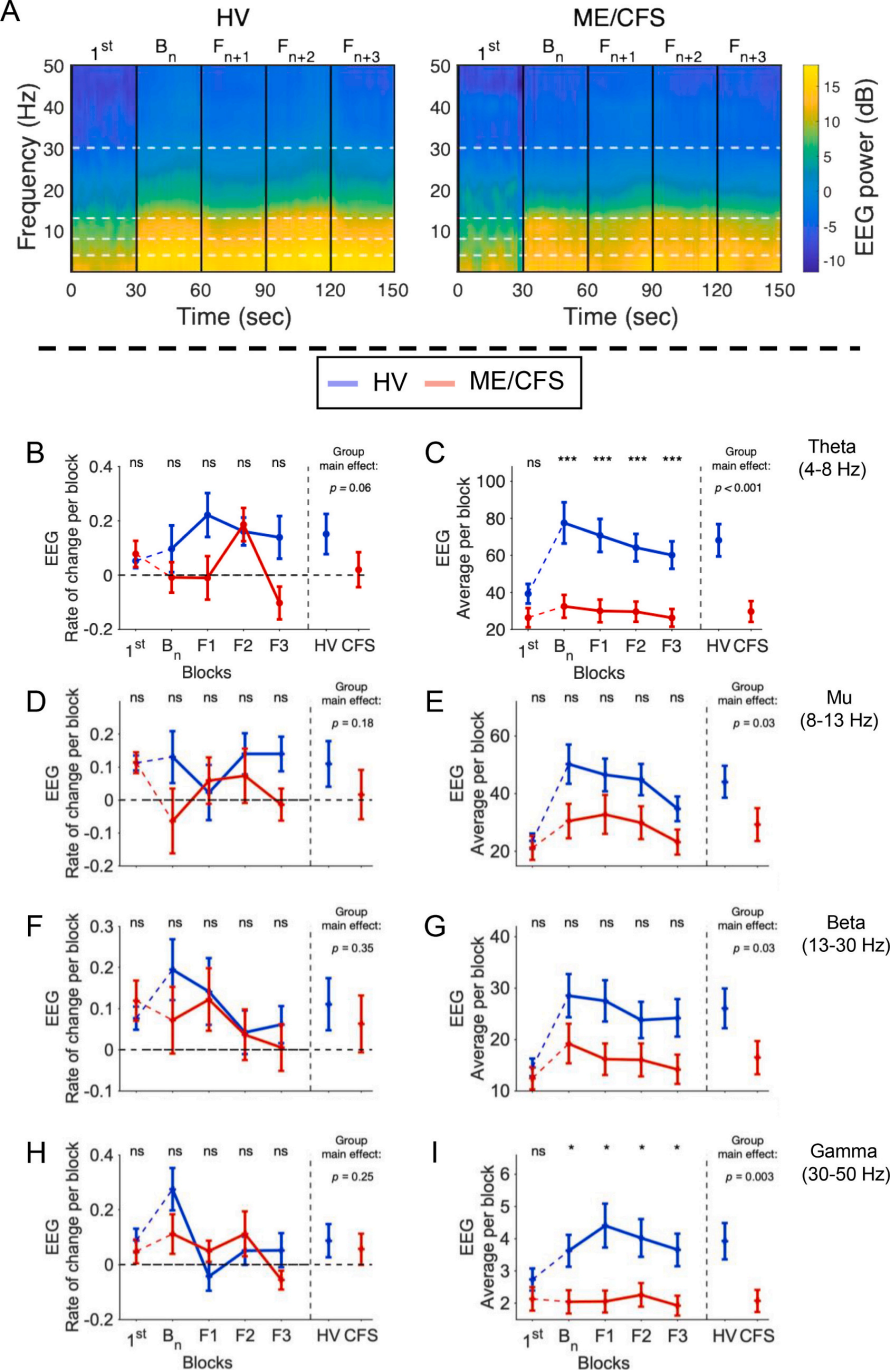
EEG power for each group in the C3 channel across the five blocks. **A:** Spectrogram for each group. Each spectrogram represents group average. The black vertical lines delineate the five blocks. The horizontal dashed white lines are at 4 Hz, 8 Hz, 13 Hz, and 30 Hz. **B-I:** EEG power for each group (means ± s.e.m.) for each of the theta, mu, beta, and gamma frequency bands. In each band, the rate of change within each block is in the left panel and the block average is in the right panel. See Fig. 3 captions for details.

The EEG spectrogram in C3 (Fig. 4A) shows that the EEG power is higher for HV than for the ME/CFS for most frequencies and blocks. Also, EEG power appeared higher in the middle of the task than at the beginning and end for both groups. Finally, EEG power is also higher around the ∼5–20 Hz range than the other frequencies for both groups.

For the slopes within blocks (Fig. 4B, D, F, and H; Table 2 for statistics details) the two-way ANOVA did not reveal any significant Groups main effect or significant interaction for any frequency band. However, in the gamma band (Fig. 4H) there was a significant Blocks main effect due to significantly higher power for the B_n_ compared to the 1st block (*t*(32) = 3.14, *p* = 0.047).

**Table 2:** Summary statistics of the two-way ANOVA of EEG power in the C3 channel.

	Groups main effect	Blocks main effect	Groups x Blocks
Slopes			
Theta Mu Beta Gamma	F(1, 34) = 3.64, *p* = 0.06	F(4, 97) = 1.42, *p* = 0.23	F(4, 97) = 2.05, p = 0.09
	F(1, 35) = 1.84, *p* = 0.18	F(4, 108) = 0.64, *p* = 0.63	F(4, 108) = 1.24, *p* = 0.29
	F(1,32) = 0.88, *p* = 0.35	F(4, 104) = 1.05, *p* = 0.38	F(4, 104) = 0.61, *p* = 0.65
	F(1,31) = 1.39, *p* = 0.24	F(4, 98) = 3.79, *p* = 0.007	F(4, 98) = 1.54, *p* = 0.19
			
Averages			
Theta Mu Beta Gamma	F(1,33) = 16.65, *p* < 0.001	F(4, 130) = 11.75, p < 0.001	F(4, 130) = 6.54, p < 0.001
	F(1, 33) = 5.16, *p* = 0.03	F(4, 130) = 17.86, p < 0.001	F(4, 130) = 3.14, *p* = 0.02
	F(1, 33) = 5.48, p = 0.03	F(4, 128) = 12.81, p < 0.001	F(4, 128) = 4.10, *p* = 0.004
	F(1, 32) = 10.05, *p* = 0.003	F(4, 125) = 5.35, *p* = 0.001	F(4, 125) = 5.01, p = 0.001

For the averages of each block (Fig. 4C, E, G, and I; Table 2 for statistics details), the Groups main effect was significant in all frequency bands, and the Groups x Block interaction was also significant in all frequency bands. First, group contrasts at the 1st block were not significantly different for any frequency band (*p* = 0.09, 0.63, 0.45, 0.25, for the theta, mu, beta, and gamma, respectively). But for each of the B_n_, F_n+1_, F_n+2,_ and F_n+3_ blocks, the HV had significantly higher power than the ME/CFS in theta and gamma bands (all *p* < 0.03), but this higher EEG power did not reach significant level in the mu and beta bands (all *p* > 0.11). The EEG power for the HV increased significantly from the 1st to the B_n_ block in all frequency bands (*p* = 0.002, 0.001, 0.001, 0.03), but for the ME/CFS it reached significant level only in the mu band (*p* = 0.10, 0.04, 0.14, 0.74). Further this increase from the 1st to the B_n_ block was significantly larger for the HV than the ME/CFS for all frequency bands (*p* = 0.002, 0.01, 0.03, 0.02).

In summary, HV had higher EEG power over C3 and a larger increase from the 1st to the B_n_ block than ME/CFS. Most of the differences between the HV and ME/CFS groups were obtained across the five blocks rather than within the blocks. This pattern was also present in the Cz and C4 channels in all four frequency bands (see Supplementary text).

The corresponding EEG data are plotted in Fig. 4B-I. *Slopes* refer to the slopes within blocks plotted in Fig. 4B-D-F-H and *Averages* refer to the averages of each block plotted in Fig. 4C-E-G-I.

### 3.6 fMRI

**Fig. 5:**
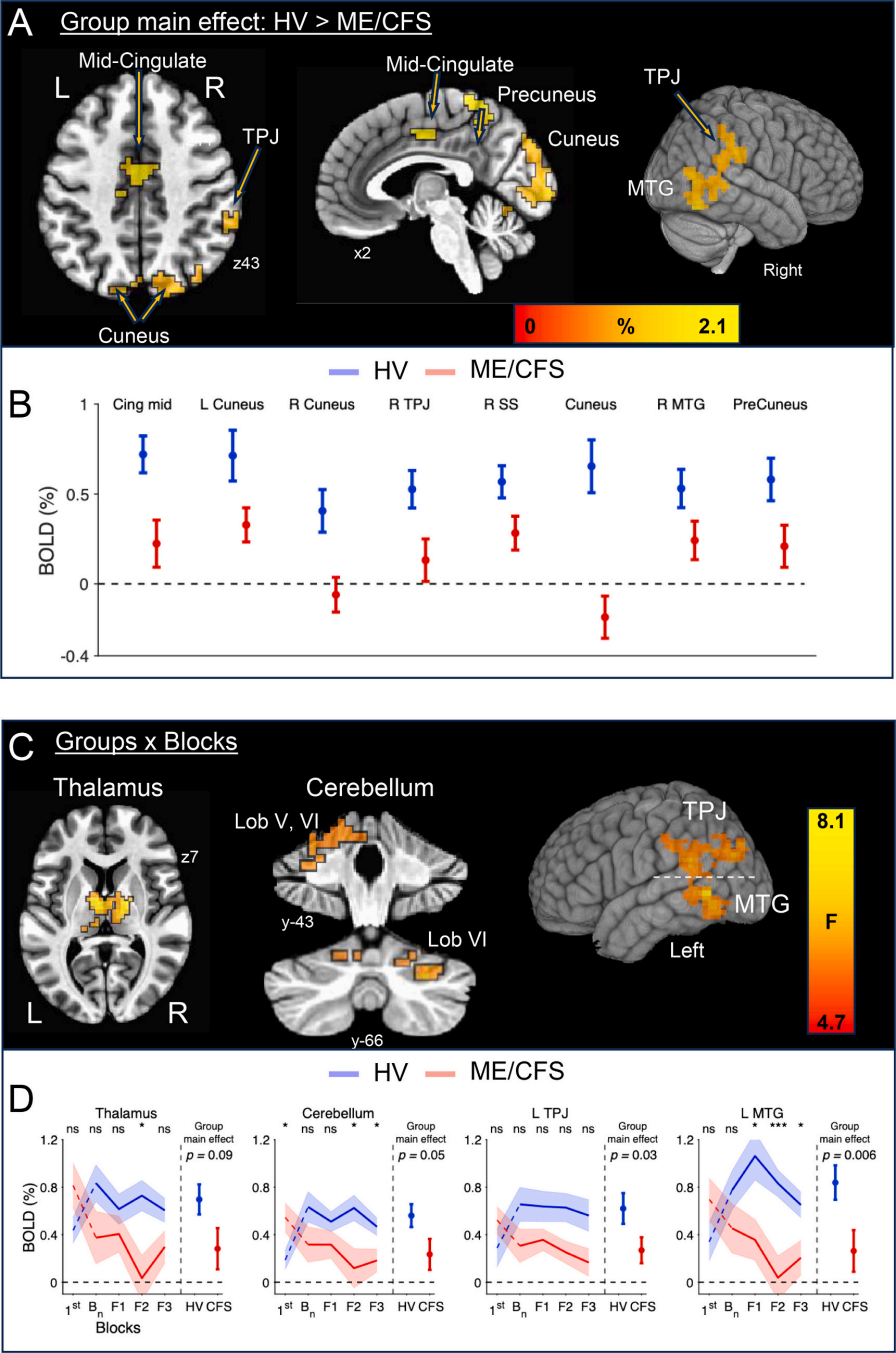
Whole-brain analysis of fMRI with a two-way Groups x Blocks ANOVA. **A:** Brain areas with significant Groups main effects. HV had higher BOLD than ME/CFS in several bilateral cortical. **B**: BOLD signal (means ± s.e.m.) of brain regions averaged across the five blocks (Table 3). **C:** Brain areas with significant Groups x Blocks interaction. Clusters included the thalamus, cerebellum, temporal-parietal junction, and middle temporal gyrus. **D**: BOLD signal (means ± s.e.m.) of these four ROI across the five blocks. F1, F2 and, F3 are the F_n+1_, F_n+2_, and F_n+3_ blocks, respectively. *L*: left, *R*: right, *TPJ*: Temporal-parietal junction, *Mid-Cingulate*: Middle-Cingulate, *MTG*: Middle Temporal Gyrus. See Fig. 3 captions for details.

Whole brain: The Groups main effect of the two-way ANOVA was significant in several bilateral areas (Fig. 5A-B; Table 3) including the cuneus, precuneus, mid-cingulate gyrus, temporal lobe, and the temporal-parietal junction (TPJ). HV had significantly higher BOLD signal than ME/CFS for all these areas. We found no areas with higher signal for ME/CFS. We show the BOLD signal for each group (averaged across blocks) for these brain regions in Fig. 5B.

**Table 3:** Brain areas with significant Groups main effects of BOLD signal.

Brain areas	MNI coordinates			*F* values	*p* values
	x	y	z		
Cingulate mid	−2	−9	44	5.59	0.02
L Cuneus	−16	−84	40	8.94	0.005
R Cuneus	14	−80	40	10.78	0.002
R TPJ	59	−44	22	11.26	0.002
R SS	40	−35	58	8.40	0.007
Cuneus	0	−87	13	11.20	0.002
R MTG	61	−50	2	4.36	0.04
L Precuneus	−4	−51	57	8.71	0.006

Information on the brain areas with significant Groups main effects from the two-way Groups x Blocks ANOVA. The *F* and *p* values are the F statistics of the Groups main effect of the two-way ANOVA and the associated *p* value. *TPJ*: temporal-parietal junction, *Cingulate mid*: Cingulate middle, *SS*: somatosensory cortex, *L*:Left, *R*:Right.

The Groups x Blocks interaction revealed two large clusters (Fig. 5C-D; Table 4). One encompassed the cerebellum (lob. V and VI) and the thalamus, and the other covered a large portion of the left hemisphere from the inferior temporal gyrus up to the inferior parietal lobe. We separated each cluster in two distinct portions because they covered separate brain regions. For the first, we isolated the thalamus by keeping the voxels that were superior to coordinate z ≥ 7 and for the cerebellum, we kept voxels inferior to z ≤ −7. For the second, we used the z = 19 to separate the cluster in two pieces (dotted line in Fig. 5C) forming a cluster centered in the middle temporal gyrus (MTG) and a cluster in the TPJ. BOLD signal is shown for each group and the five blocks in these four clusters (Fig. 5D). In all these clusters, BOLD signal was higher for HV at every block but the 1st where BOLD signal was slightly higher for MECFS. The statistical significance of the group contrasts at every block is shown in Fig. 5D. Further, BOLD signal for HV increased significantly from the 1st to the B_n_ block in each cluster (all *p* < 0.05), while for ME/CFS, BOLD signal decreased in each cluster (*p* = 0.06 in the thalamus and *p* > 0.17 for the other three clusters). This opposite direction of BOLD signal change between the 1st and the B_n_ block gave a significant group difference for each of the four clusters (all *p* < 0.01).

**Table 4:** Brain areas with significant Groups x Blocks interaction of BOLD signal.

Brain areas	MNI coordinates			*F* values	*p* values
	x	y	Z		
Thalamus	−3	−16	8	6.06	0.0002
Cerebellum (V, VI)	−7	−50	−18	6.54	0.00008
L TPJ	−53	−55	32	4.79	0.001
L MTJ	−55	−57	−5	7.30	0.00003

Information on the brain areas with significant Groups x Blocks interaction from the two-way Groups x Blocks ANOVA The *F* and *p* values are the F statistics of the Groups x Blocks interaction of the two-way ANOVA and the associated *p* value. *TPJ*: temporal-parietal junction, *MTG*: middle temporal gyrus, *L*: Left, *R*: Right.

**Fig. 6:**
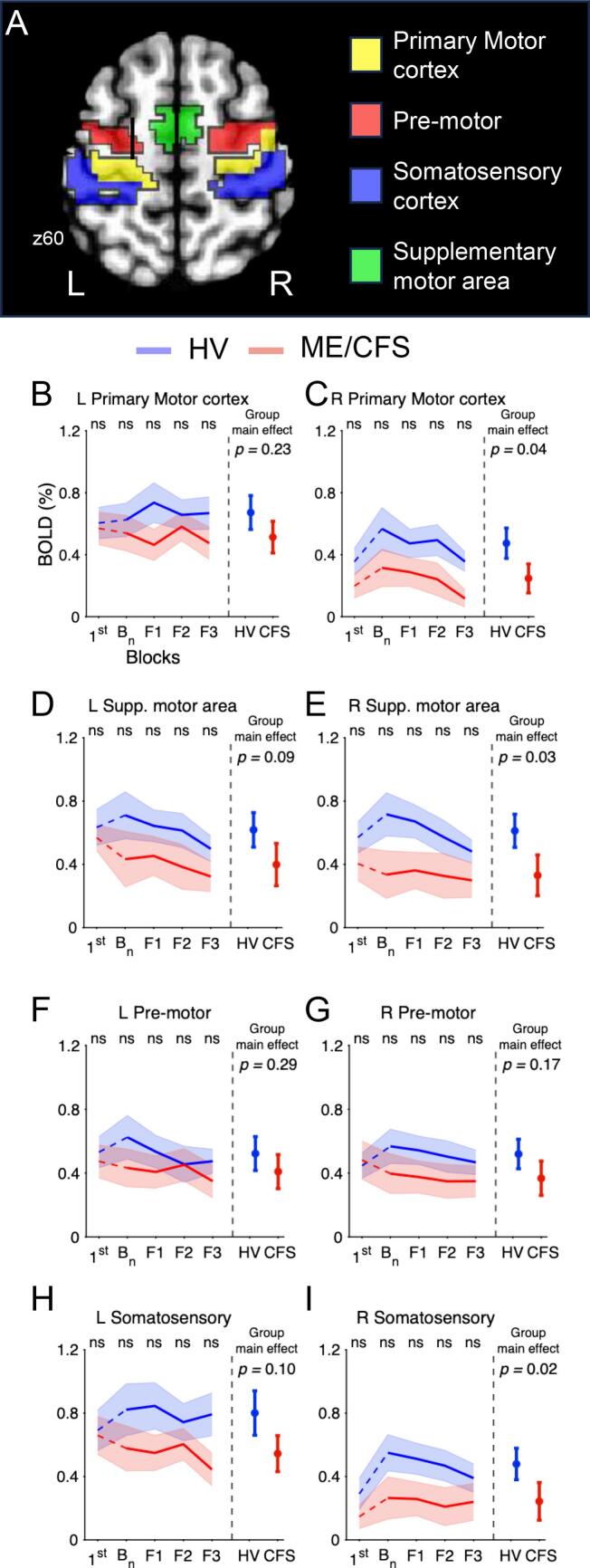
ROI analysis of fMRI with the two-way Groups x Blocks ANOVA. **A:** ROI included the bilateral primary motor cortex, pre-motor, supplementary motor area, and somatosensory cortex. **B-I**: BOLD signal across the five blocks for both groups. *L*: left, *R*: right. See Fig. 3 captions for details.

ROI: As can be seen in Fig. 6B-I, HV had higher BOLD signal than ME/CFS in all ROIs and at every block except at the 1st block where BOLD signal was almost equivalent for each group. The two-way ANOVA revealed a significant Groups main effects in the right M1, right SMA, and right somatosensory cortex (Table 5 for statistics details). There was no significant Blocks main effect and no significant interaction (*p* > 0.29 and *p* > 0.18, respectively). BOLD signal for HV increased from the 1st to the B_n_ block but not significantly (*p* > 0.12 for each ROI) followed by a slow decrease, while BOLD signal for ME/CFS decreased slowly but not significantly across the five blocks. This group difference from the 1st to the B_n_ block was not significant (all *p* > 0.23).

**Table 5:** Statistics of the two-way ANOVA for the ROI analysis of BOLD signal.

Brain areas	Groups main effects	Blocks main effects	Groups x Blocks
L M1	F(1, 34) = 1.52, p = 0.23	F(4, 134) = 0.12, *p* = 0.97	F(4, 134) = 0.84, *p* = 0.50
R M1	F(1, 34) = 4.45, *p* = 0.04	F(4, 134) = 1.67, *p* = 0.16	F(4, 134) = 0.16, *p* = 0.96
L SMA	F(1, 34) = 2.98, *p* = 0.09	F(4, 134) = 0.97, *p* = 0.43	F(4, 134) = 0.26, *p* = 0.91
R SMA	F(1, 34) = 5.17, *p* = 0.03	F(4, 134) = 0.65, *p* = 0.63	F(4, 134) = 0.48, *p* = 0.75
L PM	F(1, 34) = 1.17, *p* = 0.29	F(4, 134) = 0.53, *p* = 0.71	F(4, 134) = 0.53, p = 0.71
R PM	F(1, 34) = 1.95, *p* = 0.17	F(4, 134) = 0.38, *p* = 0.82	F(4, 134) = 0.70, *p* = 0.59
L SS	F(1, 34) = 2.86, *p* = 0.10	F(4, 134) = 0.33, *p* = 0.85	F(4, 134) = 1.09, *p* = 0.37
R SS	F(1, 34) = 5.69, *p* = 0.02	F(4, 134) = 1.56, *p* = 0.19	F(4, 134) = 0.49, p = 0.75

Statistics of the two-way ANOVA for the ROI analysis of the BOLD signal. *M1*: Primary motor cortex, *PM:* pre-motor, *SMA:* Supplementary motor area, *SS:* somatosensory cortex; *L*: Left, *R*: Right.

In summary, HV had higher BOLD signal than ME/CFS across the brain and task blocks but the 1st, and the BOLD signal in HV increased more from the 1st to the B_n_ block than for ME/CFS.

### 3.7 Corticomuscular coherence directionality

**Fig. 7:**
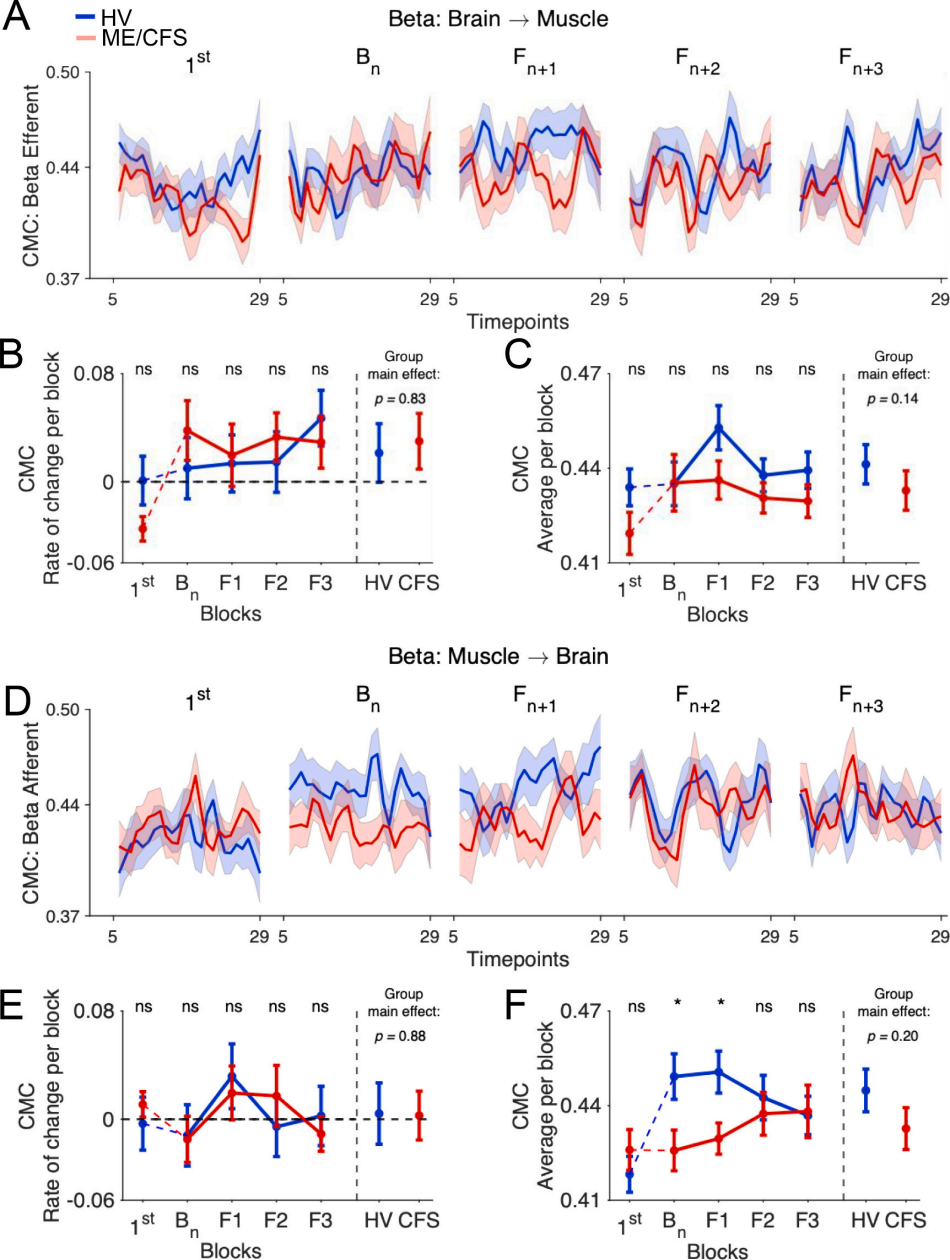
Corticomuscular coherence directionality (CMC) in the beta band (13–30 Hz) in the efferent and afferent direction between C3 and forearm muscles. All figures show means ± s.e.m. **A**: CMC in the efferent direction across all five blocks and 25 timepoints. **B:** CMC rate of change within each block. There was no group difference. **C:** CMC averages of each of the five blocks. There was no group difference. **D:** CMC in the afferent direction. **E:** CMC rate of change within each block. There was no group difference. **F:** CMC averages of each of the five blocks. HV had higher CMC than ME/CFS at the B_n_ and F_n+1_ blocks. See Fig. 3 captions for details.

Efferent direction: beta. CMC efferent in the beta band is shown for each group, blocks, and timepoints in Fig. 7A. For the slopes within blocks (Fig. 7B), the two-way ANOVA did not reveal any significant Groups main effect or interaction, but there was a significant Blocks main effect (Groups: F(1, 34) = 0.06, *p* = 0.83; Blocks: F(4, 136) = 2.45, *p* = 0.049; Interaction: F(4, 136) = 0.88, *p* = 0.48). The main effect of Blocks was due to an increase in the slope's steepness across blocks. For the averages per block (Fig. 7C), the two-way ANOVA did not reveal any significant Groups main effect or interaction, but there was a significant Blocks main effect (Groups F(1, 34) = 1.89, *p* = 0.14; Blocks: F(4, 136) = 2.59, *p* = 0.04; Interaction: F(4, 136) = 0.82, *p* = 0.51). The Blocks main effect was due to an increase from the 1st to the F_n+1_ block.

Afferent direction: beta. CMC afferent in the beta band is shown for each group, blocks, and timepoints in Fig. 7D. For the slopes within blocks (Fig. 7E), the two-way ANOVA did not reveal any significant Groups or Blocks main effect or interaction (Groups: F(1, 34) = 0.03, *p* = 0.88; Blocks: F(4, 136) = 1.17, *p* = 0.33; Interaction: F(4, 136) = 0.4, *p* = 0.81). For the averages per block (Fig. 7F), the two-way ANOVA revealed a significant interaction (Groups: F(1, 34) = 1.56, *p* = 0.20; Blocks: F(4, 136) = 4.73, *p* = 0.001; Interaction: F(4, 136) = 3.81, *p* = 0.006). Group contrast showed that HV had significantly higher CMC than ME/CFS at the B_n_ and F_n+1_ blocks (*p* < 0.05). Within group contrast showed that HV significantly increased their CMC from the 1st to the other four blocks (all *p* < 0.05), while the ME/CFS remained stable across all blocks (all *p* > 0.45).

In the gamma band, the results resembled those of the beta band (See Supplementary text).

In summary, HV had higher CMC-afferent than ME/CFS in the beta and gamma bands and HV increased their CMC-afferent across blocks in the gamma band.

### 3.8 Medication in ME/CFS

Central acting medications with long half-lives were continued for nine ME/CFS. To address the effects of taking these medications on our metrics, we directly compared the nine ME/CFS who remained on-medication with the six who did not take such medication (Supplementary text section *Influence of Medication)*. In summary, we found no instance where there was a significant group difference between those ME/CFS who took the centrally acting agents and those who did not.

## 4 Discussion

We aimed to show how the neuromuscular system of individuals with ME/CFS responded during a fatiguing task to determine whether physical fatigue, a hallmark of ME/CFS, is of central or peripheral origin.

Our most novel results relate to the lack of neuromuscular adaptation in ME/CFS compared to the HV. ME/CFS did not change their muscular and brain activity as measured via DI, EEG, BOLD, and CMC, and as a result, they demonstrated fatigue earlier than HV as shown by the earlier occurrence of the B_n_ block and the steeper decline of grip force across the five task blocks. In fact, these neuromuscular metrics in ME/CFS remained stable or showed only slight changes across the five task blocks. By contrast, HV showed drastic changes in their neuromuscular system during and across the task blocks by increasing their muscular and brain activity (DI, EEG, BOLD, CMC) and these seemed to delay the onset of performance decline. Further, these neuromuscular changes occurred even before the grip force level began to decrease. Early fatigue detection can prevent accidents in real-world settings (Zhang et al., 2023). Both ME/CFS and HV felt more fatigued after the task, but ME/CFS always felt more fatigued than HV.

During the 1st and B_n_ blocks, grip force was indistinguishable between the HV and the ME/CFS, as expected; this reduces the possibility of being a confound for the interpretation of the other metrics. Further, this similar grip force level for each group during the 1st and B_n_ blocks and during the MVC suggest that the earlier onset of performance decline in ME/CFS is not related to a reduction in general strength (Charlton et al., 2025).

The HV maintained a steady grip force level during the 1st and B_n_ blocks, but still they showed a steady increase in DI. The ME/CFS also maintained a steady grip force level, but their DI decreased and was lower than the HV's. The increase of DI is a hallmark of muscular fatigue and indicates a shift of the EMG power spectrum from high to low frequencies caused by a decrease in the muscle fiber conduction velocity (Bigland-Ritchie et al., 1981; De Luca, 1984; Dimitrov et al., 2006; Dimitrova et al., 2005; Wan et al., 2017; Zhao et al., 2022a). The lack of a DI increase in ME/CFS suggests that they did not engage in the task enough to develop muscular fatigue. Alternatively, the lack of a DI increase could relate to muscles deficiencies as often reported in this disorder (Rutherford et al., 2016; Syed et al., 2025; Wang et al., 2023). But this is less likely since the ME/CFS and HV generated similar EMG amplitude as measured with RMS. Further, in our prior work, with eleven of the current fifteen ME/CFS participants, we did not find evidence of impaired muscles fiber composition (Walitt et al., 2024). It remains uncertain why DI at block B_n_ did not increase in ME/CFS while they generated similar force level as the HV. Thus, the muscle deficiencies in ME/CFS may not be the only or the most essential factor explaining their early onset of performance decline.

Brain activity in HV as revealed with EEG power and BOLD signal increased from the 1st to the B_n_ block in many cortical and cerebellar areas, while in ME/CFS brain activity remained unchanged or showed only small fluctuations. The absence of an increase in brain activity in ME/CFS suggests that their early decline in performance is of central origin. This lack of increase of brain activity in ME/CFS could explain the lack of DI increase at the B_n_ block. Prior work with transcranial magnetic stimulation over the primary motor cortex also suggested a central origin of fatigability in ME/CFS (Sacco et al., 1999; Samii et al., 1996; Schillings et al., 2006; Starr et al., 2000). We found these group differences in the contralateral but also in the ipsilateral hemisphere (Liu et al., 2007). The ipsilateral involvement would allow for the recruitment of other portions of the brain to maintain optimal performance and would occur via the ipsilateral projections (Dum and Strick, 1996). This absence of ipsilateral recruitment in ME/CFS further suggests a central origin for their early performance decline.

Prior work in HV reported fatigability-related increases of EEG power in various brain areas and frequencies (mostly 8–30 Hz range approximately) (Bedard et al., 2025; Berchicci et al., 2013; Enders et al., 2016; Guo et al., 2014; Johnston et al., 2001; Schillings et al., 2006; Suviseshamuthu et al., 2022; Wang et al., 2017). Here, we found group differences in the C3, C4, and Cz electrodes, which overlay the bilateral cortical sensorimotor areas, and across the 4–50 Hz range. EEG power is greatly influenced by the degree of neuronal synchronization (Nunez and Srinivasan, 2006), so the increase in EEG power in HV could indicate that the sensorimotor cortex was recruiting additional motor units and/or increasing the firing rate of the already active motor units to delay the onset of physical fatigue. This recruitment became more synchronized and increased EEG power with fatigability. This description is in line with the increase of DI in HV. On the other hand, because EEG power can be higher at rest than during active tasks (Nunez and Srinivasan, 2006), the increase in EEG power could also relate to some form of adaptation. In ME/CFS, EEG power changed only slightly, which probably prevented recruitment of muscles as seen in HV. Therefore, DI did not increase, and the occurrence of the B_n_ block and the decline in performance occurred earlier than for the HV.

HV also showed increases of BOLD signal in the cerebellum, sensorimotor cortical brain areas, mid-cingulate gyrus, and TPJ, while ME/CFS showed a slight decrease. Prior work in HV showed an involvement of these areas during fatiguing tasks like ours (Benwell et al., 2007; Herath et al., 2017; Jiang et al., 2016; Post et al., 2009). As with EEG, the lack of an increase of the BOLD signal in ME/CFS and the absence of ipsilateral recruitment suggest a central origin for the earlier performance decline. Alternatively, this lack of BOLD signal adaptation might relate to a reduced cerebral blood flow due to an abnormal neurovascular coupling that has been reported in ME/CFS (Shan et al., 2018). However, ME/CFS had a similar and even slightly higher BOLD signal than HV at the 1st block in many brain areas (Fig. 5D and 6). It thus seems unlikely that the lack of BOLD signal increase in ME/CFS can fully be explained by reduced cerebral blood flow.

Regarding CMC, we did not find group differences for CMC-efferent. But CMC-afferent increased in HV from the 1st to the B_n_ block but remained flat in ME/CFS. CMC directionality reflects directed and rhythmic electrical activity to and from the motor cortical areas and the contralateral muscles during movement. This study is the first to report fatigability-related CMC in ME/CFS. CMC in HV during fatigability is still controversial, as some studies show increases and others decreases (see *Introduction*). Since CMC-efferent for ME/CFS was like that of HV, it may indicate an intact information flow from the sensorimotor areas to the muscles in ME/CFS. The higher CMC-afferent in HV compared to ME/CFS could relate to the higher DI, which reflects muscular fatigue, and this could have increased the flow of afferent information to update the brain about the muscle's state. This flow of afferent information may be mediated via the afferent muscle fibers III/IV that carry information about the mechanical and biochemical properties of the muscles and relay that information to the brain and also the motoneurons in the spinal cord (Amann et al., 2020; Laurin et al., 2015). The lower CMC-afferent in ME/CFS is probably related to the absence of a DI increase.

The blocks F_n+1_, F_n+2_, and F_n+3_ represent the onset and the continuance of the performance decline for each group. ME/CFS participants reached the F_n+1_ block much earlier and generated less grip force during the F_n+2_ and F_n+3_ blocks than the HV. In ME/CFS, the neuromuscular responses (DI, EEG, BOLD, and CMC) basically remained at the same level as in the earlier 1st and B_n_ blocks or even slightly decreased as shown for EEG power and BOLD signal. This reduced activity could relate in part to the lower level of grip force generated by the ME/CFS and not just to the ME/CFS disorder. In HV, the neuromuscular responses, which had peaked at the B_n_ block for almost all metrics (a few peaked at the F_n+1_ block), plateaued and started to gradually decrease. These changes in HV could indicate a return to a more homeostatic state in the brain, muscles, and body to prevent a catastrophic depletion of resources and avoid harms due to exertion (Hureau et al., 2018; Mosso, 1915; Noakes, 2012).

Put together, these results show that the ME/CFS had limited engagement of their neuromuscular system that was substantially less than what was observed in HV. This resulted in an early decline in performance, and thus, an early onset of physical fatigue; although, we should be careful before labeling this as fatigue since their neuromuscular system showed much reduced engagement. Importantly, this lack of engagement is not conscious or deliberate. Several factors may explain this lack of engagement including, but not limited, to the fear, conscious or unconscious, of triggering post-exertional malaise (Stussman et al., 2020), musculoskeletal/joint pain (Rutherford et al., 2016), or elevated perceived exertion (Barhorst et al., 2020).

Musculoskeletal pain can prevent or limit someone from generating force for a long time resulting in early fatigue. Brain areas involved in painful experience, such as the amygdala, insula, and anterior cingulate, can communicate with the motor system and modulate the motor output. Administering a measure to understand task-related pain could improve understanding of musculoskeletal pain on physical engagement in ME/CFS. Changing brain activity in pain-related areas via brain stimulation or medication could potentially improve physical engagement. A better understanding of the impact that various medication have on these areas could also be beneficial; however, the use of central-acting medications commonly used for pain disorders did not substantially change these results. Perceived exertion, which was not measured in this study, could also affect motor output, via a different brain network than pain, probably including prefrontal areas. Connectivity analyses could help better understand how these areas communicate with the motor system and how they affect behavior.

While we have interpreted the ME/CFS results as evidence of a central origin of fatigue, our methods (e.g., task design, neuroimaging) cannot establish a direct causation between brain and muscles responses and central fatigue. Our methods allowed us to show an association. The use of a direct intervention such as TMS or other brain stimulation would be more able to address causation of fatigue, albeit with a small number of potential brain areas to target. We have used our results to *suggest* a central origin as it seemed the most likely explanation. But the ME/CFS disorder affects several brain and body processes that may in turn affect brain areas involved in generating movements and behavior; we have shown the latter.

Limitations. The most important limitation of our work relates to the low number of participants. Other limitations include the heterogeneity of the ME/CFS diagnosis and the two different MRI protocols for the HV. While we have included a covariate to account for the two different MRI protocols, it remains an extra source of variability. ME/CFS with depression or psychiatric disorders were not included. Thus, our results may not generalize to ME/CFS with these disorders. Medication with centrally acting effects (e.g., SSRI/SNRIs) were continued during the study for nine ME/CFS, but the results of these nine ME/CFS were not significantly different than the other six ME/CFS who did not take such medication (Supplementary text section *Influence of Medication*). Some results for eleven of the fifteen ME/CFS including grip force, DI, and fMRI were reported in our previous work (Walitt et al., 2024), so they are not completely independent of prior findings. We did not collect the VAS data for some participants, but this occurred in equal proportion in each group, 6/19 for HV and 5/15 for ME/CFS, 68.5% and 66.6%, respectively. We only collected one type of subjective measure during the experiment. Adding measure of perceived exertion or task difficulty could have helped better explain our results.

## 5 Conclusion

In healthy individuals, fatigability is the objective decline of performance over time. Fatigability in ME/CFS should be interpreted differently. While our results suggested that ME/CFS fatigability has a central origin, we would argue that since the fatigability process in ME/CFS did not fully take place, at least not like it did in HV, it may have an earlier origin than the neuromuscular system. The pathophysiology of ME/CFS is marked by a cascade of events affecting the autonomic, neuroendocrine, immunologic, bioenergetic, and physiologic systems (Hornig et al., 2015; Missailidis et al., 2019; Nelson et al., 2019; Paul et al., 2021). These processes are most likely the source for the lack of engagement of the neuromuscular system (Walitt et al., 2024).

## CRediT authorship contribution statement

**Patrick Bedard:** Writing – review & editing, Writing – original draft, Visualization, Validation, Methodology, Formal analysis. **Kristine M. Knutson:** Writing – review & editing, Methodology, Investigation. **Patrick M. McGurrin:** Writing – review & editing, Writing – original draft, Methodology, Investigation. **Felipe Vial:** Writing – original draft, Methodology, Conceptualization. **Traian Popa:** Writing – original draft, Methodology. **Silvina G. Horovitz:** Writing – review & editing, Writing – original draft, Investigation, Formal analysis, Conceptualization. **Mark Hallett:** Writing – original draft, Project administration, Methodology, Investigation, Conceptualization. **Avindra Nath:** Investigation, Funding acquisition, Conceptualization. **Brian Walitt:** Writing – original draft, Project administration, Methodology, Investigation.

## Consent for publication

Not applicable.

## Ethics approval and consent to participate

All participants provided informed consent in accordance with the Institutional Review Board of the National Institutes of Health and the Declaration of Helsinki.

## Funding

This research was supported in part by the Intramural Research Program (ZIA NS003157) of the National Institutes of Health (NIH), National Institute of Neurological Disorders and Stroke (NINDS).

## Declaration of competing interest

The authors declare that they have no known competing financial interests or personal relationships that could have appeared to influence the work reported in this paper.

## Data Availability

Data will be made available on request.
