# Supplementary material for: Central origin of fatigability in Myalgic encephalomyelitis/chronic fatigue syndrome revealed by multimodal neuroimaging
Source: Neuroimage Clin. 2026 Jul 28;51:104041. doi: 10.1016/j.nicl.2026.104041 (PMC13471709; doi:10.1016/j.nicl.2026.104041)

Supplementary Figure 1

RMS of EMG

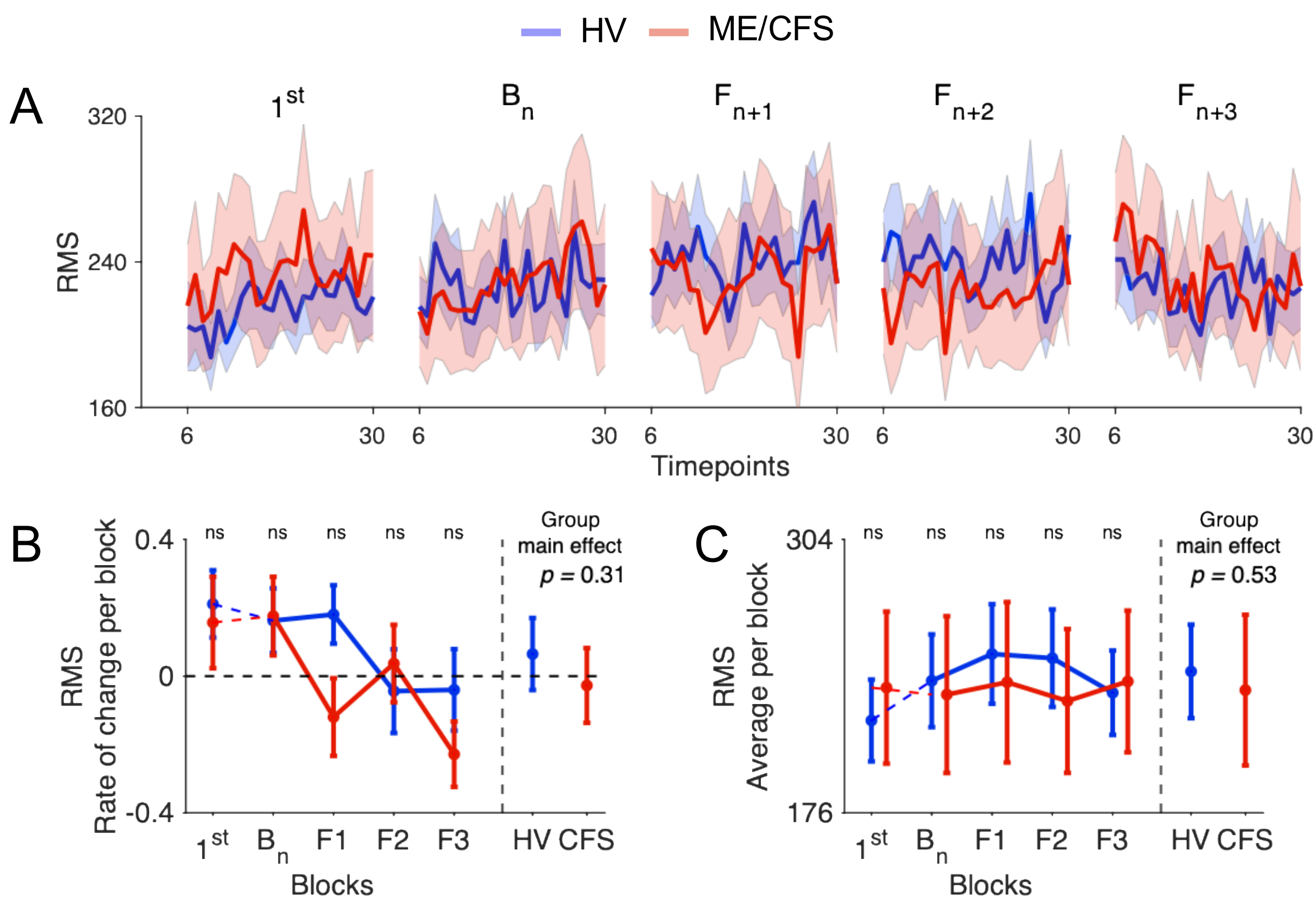

Supplementary Figure 2

EEG: Channel C4

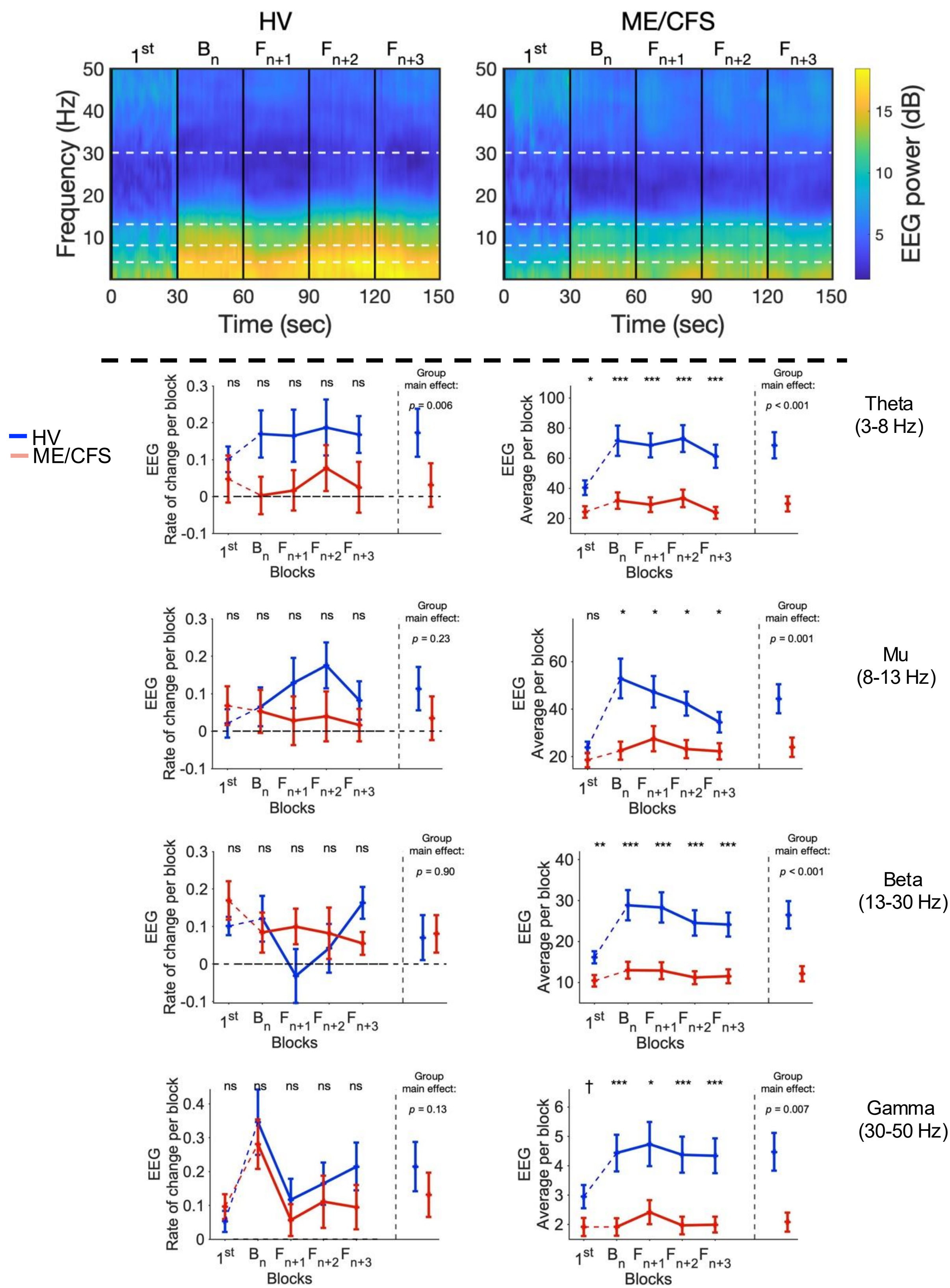

Supplementary Figure 3

EEG: Channel Cz

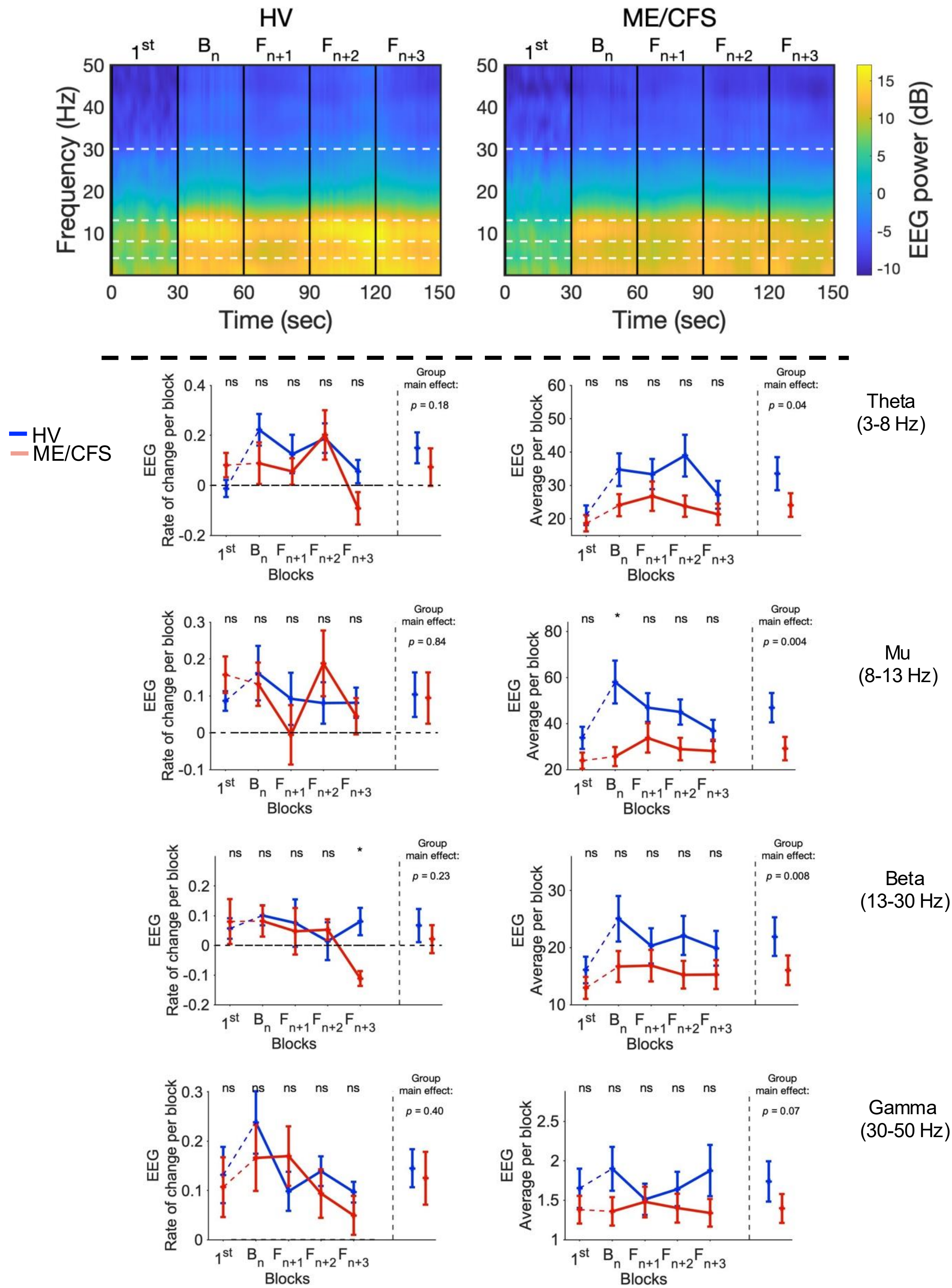

Supplementary Figure 4

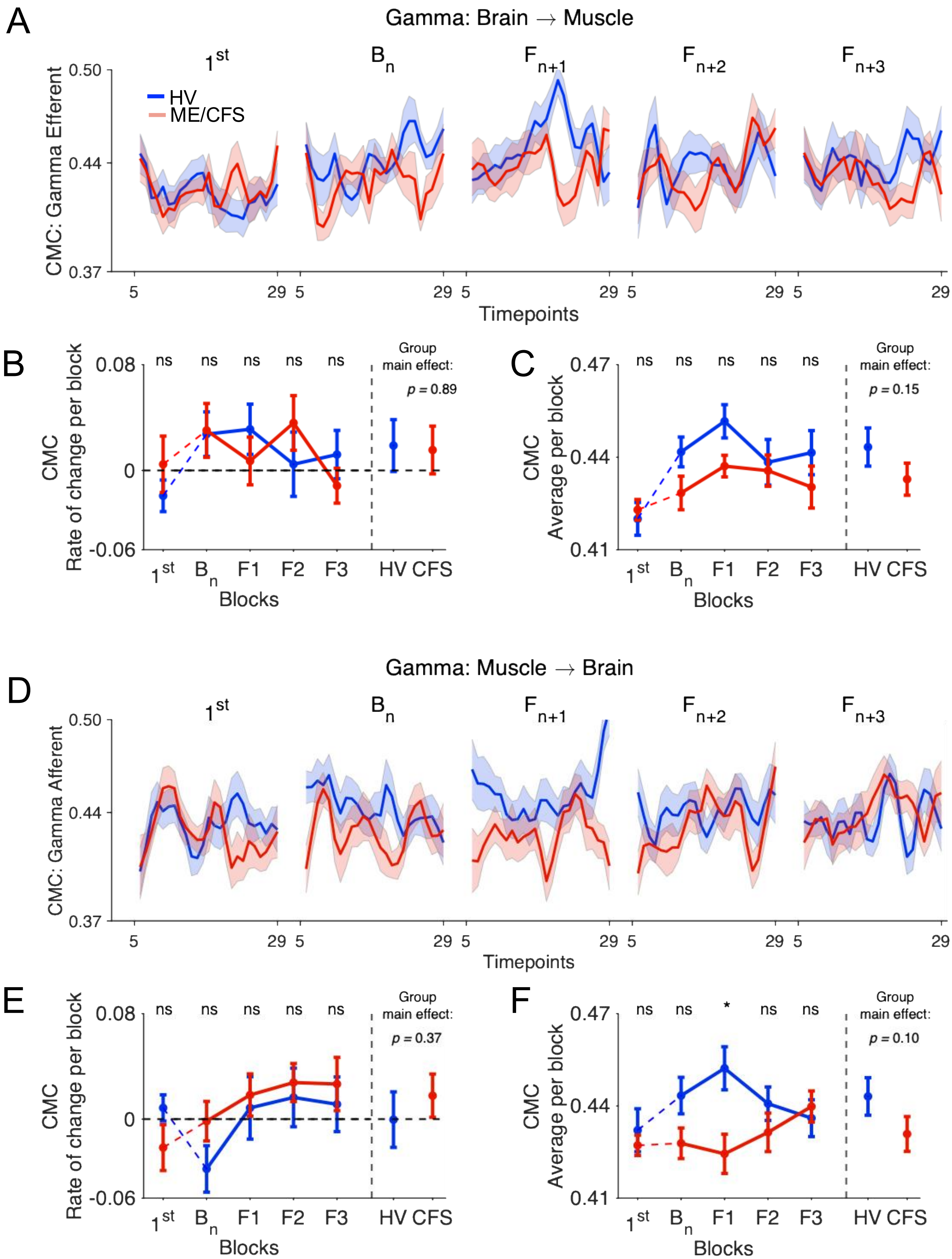

Supplement: Supplementary file 1 [file mmc1.pdf]
