# Supplementary material for: Central origin of fatigability in Myalgic encephalomyelitis/chronic fatigue syndrome revealed by multimodal neuroimaging
Source: Neuroimage Clin. 2026 Jul 28;51:104041. doi: 10.1016/j.nicl.2026.104041 (PMC13471709; doi:10.1016/j.nicl.2026.104041)
Supplement: Supplementary file 2 [file mmc2.docx]

Table 1. Participants demographics

|  | Age | Sex | Education |
| --- | --- | --- | --- |
| Participants |  |  |  |
| HV |  |  |  |
| 1 | 42 | M | 4 |
| 2 | 61 | F | 2 |
| 3 | 20 | F | 2 |
| 4 | 54 | F | 4 |
| 5 | 21 | M | 3 |
| 6 | 56 | M | 1 |
| 7 | 36 | M | 3 |
| 8 | 53 | F | 4 |
| 9 | 40 | M | 3 |
| 10 | 36 | M | 4 |
| 11 | 26 | M | 4 |
| 12 | 53 | M | 3 |
| 13 | 42 | M | 4 |
| 14 | 51 | M | 4 |
| 15 | 38 | F | 4 |
| 16 | 34 | F | 4 |
| 17 | 58 | F | 4 |
| 18 | 61 | M | 4 |
| 19 | 56 | M | 4 |
|  |  |  |  |
| ME/CFS |  |  |  |
| 1 | 49 | M | 4 |
| 2 | 51 | F | 2 |
| 3 | 46 | M | 2 |
| 4 | 38 | F | 4 |
| 5 | 44 | F | 3 |
| 6 | 19 | F | 2 |
| 7 | 37 | M | 4 |
| 8 | 26 | F | 3 |
| 9 | 43 | M | 4 |
| 10 | 29 | M | 3 |
| 11 | 32 | M | 3 |
| 12 | 55 | F | 3 |
| 13 | 27 | M | 2 |
| 14 | 44 | F | 3 |
| 15 | 53 | M | 2 |
|  | *p* = 0.29 | *p* = 0.56 | *p* = 0.11 |

Education: 0: less than high school, 1: high school, 2: some college, 3: college graduate, 4: advanced degree.
