# Supplementary material for: Central origin of fatigability in Myalgic encephalomyelitis/chronic fatigue syndrome revealed by multimodal neuroimaging
Source: Neuroimage Clin. 2026 Jul 28;51:104041. doi: 10.1016/j.nicl.2026.104041 (PMC13471709; doi:10.1016/j.nicl.2026.104041)
Supplement: Supplementary file 3 [file mmc3.docx]

**Additional Table 1. HV and ME/CFS medication**

| HV | Medication | |  |
| --- | --- | --- | --- |
| 1 | Vitamin D3 | |  |
| 2 | None | |  |
| 3 | None | |  |
| 4 | Magnesium, Hormone replacement therapy, aspirin 81mg, Vitamins | |  |
| 5 | None | |  |
| 6 | Iron with Vitamin C, Zinc | |  |
| 7 | Fish oil | |  |
| 8 | Mirena IUD | |  |
| 9 | None | |  |
| 10 | None | |  |
| 11 | None | |  |
| 12 | None | |  |
| 13 | ranitidine, pantoprazole | |  |
| 14 | Hydrochlorothiazide, lisinopril, multivitamin | |  |
| 15 | Nexplanon, Magnesium, Coenzyme Q, multivitamin, Vitamin B complex, Vitamin D | |  |
| 16 | Rizatripan PRN | |  |
| 17 | multivitamin, vitamin C, zinc, collagen | |  |
| 18 | Lion's mane supplement, Bio complete supplement, morning complete supplement | |  |
| 19 | None | |  |
|  |  | |  |
| ME/CFS | Medication |  |  |
| 1 | Clonazepam 0.5 mg, Vicodin 5/325mg, Metamucil 2 tsp, Polyethylene Glycol 3350 Powder for Oral Solution (17 gram packet), Psyllium Powder 14 ounce container (3.4 grams), Ranitidine 150 mg, Probiotics, CoQ10, Zinc, L-carnitine, B Vitamins, Magnesium, Mushrooms, Roots, D-ribose, Selenium, Vitamin D | |  |
| 2 | Clonazepam 1 mg tablet, Ferrous Sulfate 325 mg tablet, Gabapentin 100 mg capsule, Gabapentin 300mg, Metoprolol Tartrate 25mg, Perfect biotics 30billion CFU's, 1 capsule, Sertraline 50 mg, Sertraline 150mg, Valtrex 1 gm, Zolpidem 10mg, Zolpidem 5mg, Boric acid topical suppository 600mg, Norgestrel/Ethinyl Estradiol 0.3mg/0.03mg | | |
| 3 | Valacyclovir (VALTREX) 1000mg, Valtrex 1000mg, Cetirizine 10 mg tablet, FluocinoNIDE Cream 0.05%, Loperamide 2 mg, Fexofenadine 180 mg, Allegra 160mg, Clonazepam 0.5 mg, Ketoconazole Shampoo 2%, Desonide Cream 0.05%, Propranolol HCL 10 mg, Propanolol 5mg, Gabapentin 300mg, Liver efficiency supplement 1 capsule, Magnesium capsule 500 mg 2 capsules, Naltrexone 4.5 mg, Ranitidine 150mg, Sodazyme 100mg 3 capsules, Testosterone cream 2 pumps, Testosterone 50mg (10%) gel 2 pumps, Ubiquinol QH 100mg, B12 Methyl 12.5 mg/ ml (0.8mL), Cimetadine tab 200 mg, | | |
| 4 | Camila (Birth control pill) 0.35 mg | | |
| 5 | K2-D3 90mcg/5000IU, Naltrexone 4.5mg, Nature Thyroid 32.5mg, Nortrel contraceptive pill 1 tab | | |
| 6 | Terbinafine 250mg | | |
| 7 | Adderall XR 15mg, Amphetamine Racemic Mix Extended Release 15 mg, Armodafinil 250 mg, Atorvastatin 10 mg, Bupropion Extended Release (XL) 150 mg, Escitalopram 20 mg, Multivitamin 1 tab, MVI 1 tab | |  |
| 8 | Allegra 180mg, Diclofenac 50mg, Mestinon 30mg, Retasis 0.05%, Singulair 10mg, Ondansetron Oral Disintegrating 4 mg tablet, Mierna IUD, Midodrine 2.5 mg ½ tab, Propranolol HCL 10mg, Liquid IV hydration multiplier | |  |
| 9 | Diphenhydramine 25mg, Fluorometholone Opth Susp 0.1%, Fluorometholone Opth Susp 0.1%, Vitamin D3 1000 IU, Vitamin B 100 complex, Zinc citrate 50mg, Neuro-Mag 144mg, Omega-3 polyunsaturated fatty acids 1200mg, Clotrimazole Cream 1%, Fish oil 1200mg, Probiotic, Selenium | |  |
| 10 | Cyclobenzaprine 10mg, Diazepam 2.5mg, Fludrocortisone 0.2mg, Fludrocortisone Acetate 100 mcg, Melatonin 6mg Naltrexone 4.5mg, Pyridostigmine 60 mg, Valcyclovir 1000mg, Propanolol HCL 10mg, Ammonium Lactate Lotion 12% | |  |
| 11 | No medications listed | |  |
| 12 | Pristiq 25 mg, progesterone 100 mg, olestric gel, testosterone gel | |  |
| 13 | No medications listed | |  |
| 14 | Introvale, buproprion 300 mg, citalopram 20 mg | |  |
| 15 | trazadone 50 mg, Cymbalta 60 mg, Gabapentin 300 mg, herbs (unknown) | |  |
|  |  | |  |
