# Supplementary material for: Central origin of fatigability in Myalgic encephalomyelitis/chronic fatigue syndrome revealed by multimodal neuroimaging
Source: Neuroimage Clin. 2026 Jul 28;51:104041. doi: 10.1016/j.nicl.2026.104041 (PMC13471709; doi:10.1016/j.nicl.2026.104041)
Supplement: Supplementary file 4 [file mmc4.docx]

**EEG in the C4 and Cz channels**

The text describes the EEG in the Cz and C4 channels shown in Supplementary Figure 2-3.

The EEG pattern shown in Figure 4 was also present in the Cz and C4 channels in all four frequency bands. However, it is important to note that the level of statistical significance, i.e. the *p*-value, of the two-way ANOVA for each of the Group main effect and Group x Blocks interaction as well as the group contrasts at each block was slightly different in those two channels, frequency bands, and for the slopes and averages per block, than in the C3 channel. But overall, the pattern of data obtained in C3 repeated itself in the Cz and C4 channels.

**Supp. Table 2. Summary statistics of the two-way ANOVA of EEG power in the C4 channel**

|  | Group main effect | Blocks main effect | Group x Blocks |
| --- | --- | --- | --- |
| Slopes |  |  |  |
| Theta  Mu  Beta  Gamma | F(1, 32) = 8.55, p = 0.006 | F(4, 104) = 0.25, p = 0.911 | F(4, 104) = 0.31, p = 0.873 |
|  | F(1, 38) = 1.50, p = 0.228 | F(4, 112) = 0.40, p = 0.81 | F(4, 112) = 1.00, p = 0.409 |
|  | F(1, 37) = 0.02, p = 0.899 | F(4, 110) = 0.98, p = 0.422 | F(4, 110) = 1.54, p = 0.196 |
|  | F(1, 26) = 2.50, p = 0.126 | F(4, 94) = 3.63, p = 0.009 | F(4, 94) = 0.51, p = 0.726 |
|  |  |  |  |
| Averages |  |  |  |
| Theta  Mu  Beta  Gamma | F(1, 33) = 19.38, p < 0.001 | F(4, 130) = 10.49, p < 0.001 | F(4, 130) = 3.84, p = 0.006 |
|  | F(1, 33) = 12.31, p = 0.001 | F(4, 129) = 13.1, p < 0.001 | F(4, 129) = 6.23, p < 0.001 |
|  | F(1, 33) = 21.38, p < 0.001 | F(4, 129) = 11.27, p < 0.001 | F(4, 129) = 5.13, p = 0.001 |
|  | F(1, 33) = 8.41, p = 0.007 | F(4, 127) = 7.21, p < 0.001 | F(4, 127) = 5.01, p = 0.001 |

*Slopes* refer to the slopes within blocks and *Averages* refer to the averages of each block

**Supp. Table 3. Summary statistics of the two-way ANOVA of EEG power in the Cz channel**

|  | Group main effect | Blocks main effect | Group x Blocks |
| --- | --- | --- | --- |
| Slopes |  |  |  |
| Theta  Mu  Beta  Gamma | F(1, 36) = 1.86, p = 0.182 | F(4, 105) = 3.75, p = 0.007 | F(4, 105) = 1.60, p = 0.18 |
|  | F(1, 29) = 0.04, p = 0.839 | F(4, 92) = 0.74, p = 0.564 | F(4, 92) = 0.76, p = 0.55 |
|  | F(1, 36) = 1.48, p = 0.232 | F(4, 109) = 0.98, p = 0.421 | F(4, 109) = 1.38, p = 0.24 |
|  | F(1, 23) = 0.74, p = 0.398 | F(4, 91) = 1.8, p = 0.136 | F(4, 91) = 0.34, p = 0.85 |
|  |  |  |  |
| Averages |  |  |  |
| Theta  Mu  Beta  Gamma | F(1, 33) = 4.53, p = 0.041 | F(4, 130) = 13.85, p < 0.001 | F(4, 130) = 3.53, p = 0.009 |
|  | F(1, 33) = 9.53, p = 0.004 | F(4, 128) = 6.23, p < 0.001 | F(4, 128) = 4.41, p = 0.002 |
|  | F(1, 33) = 7.86, p = 0.008 | F(4, 127) = 8.20, p < 0.001 | F(4, 127) = 1.76, p = 0.14 |
|  | F(1, 33) = 3.53, p = 0.069 | F(4, 122) = 1.97, p = 0.104 | F(4, 122) = 1.88, p = 0.12 |

*Slopes* refer to the slopes within blocks and *Averages* refer to the averages of each block

**CMC- gamma band**

The text describes the Corticomuscular (CMC) in the gamma band shown in Supplementary Figure 4

Efferent direction. For the slopes within blocks (see Additional Figure 4) the two-way ANOVA did not reveal any significant Groups or Blocks main effect or interaction (Groups: F(1, 34) = 0.01, *p* = 0.89; Blocks: F(4, 136) = 1.91, *p* = 0.11; Interaction: F(4, 136) = 1.14, *p* = 0.34). For the averages per block, the two-way ANOVA did not reveal any significant Groups main effect or interaction, but there was a significant Blocks main effect (Groups: F(1, 34) = 1.47, *p* = 0.15; Blocks: F(4, 136) = 5.75, *p* = 0.0003; Interaction: F(4, 136) = 0.45, *p* = 0.77). The main effect of Blocks was due to an increase from the 1^st^ to the F_n+1_ block. Within group contrast showed that HV significantly increased their CMC from the 1^st^ to the other four blocks (all *p* < 0.05), while the ME/CFS remained stable across all blocks (all *p* > 0.22).

Afferent direction. For the slopes within blocks (see Additional Figure 4) the two-way ANOVA did not reveal any significant Groups or Blocks main effect or interaction (Groups: F(1, 34) = 0.83, *p* = 0.37; Blocks: F(4, 136) = 2.13, *p* = 0.09; Interaction: F(4, 136) = 0.97, *p* = 0.43). For the averages per block, the two-way ANOVA revealed a significant interaction (Groups: F(1, 34) = 2.33, *p* = 0.10; Blocks: F(4, 136) = 1.51, *p* = 0.20; Interaction: F(4, 136) = 3.29, *p* = 0.01). The HV had a significantly higher CMC at the F_n+1_ block (*p* = 0.03; note that *p* = 0.09 at the B_n_ block).

**Influence of Medication**

We analyzed the effects of the centrally acting medications (SSRI/SNRI) taken by 9 of the 15 ME/CFS participants. We directly compared the 2 sub-groups hereafter labelled ME/CFS-on and ME/CFS-off with independent *t*-test on each of the five blocks and corrected for multiple comparisons with FDR. Thus, it refers to the data of the ME/CFS group presented in Figure 2-7, i.e. the red lines/dots, divided between the ME/CFS-on and ME/CFS-off.

**Behavioral performance**

For MVC, there were no significant difference between the ME/CFS-on and ME/CFS-off (23.2 ± 9 vs. 30.4 ± 6, *t*(13) = 1.72, *p* = 0.11).

For the occurrence of the B_n_ block, there was no significant difference between the ME/CFS-on and ME/CFS-off (5.3 ± 4 vs. 4.3 ± 5, *t*(13) = 0.44, *p* = 0.67).

For the rate of grip force decline across the 5 blocks, there was no significant difference between the ME/CFS-on and ME/CFS-off (-5.7 ± 4 vs. –6.8 ± 3, *t*(13) = 0.56, *p* = 0.58).

For the grip force slope in each of the 5 blocks, there was no significant difference between the ME/CFS-on and ME/CFS-off (*p* = 0.32, 0.60, 0.64, 0.64, 0.63 across the five blocks).

For the grip force average in each of the 5 blocks, there was no significant difference between the ME/CFS-on and ME/CFS-off (*p* = 0.41, 0.35, 0.35, 0.35, 0.41 across the five blocks).

**Subjective fatigue evaluation**

For the VAS, there was no significant difference between the ME/CFS-on and ME/CFS-off on for the mental or physical VAS (*p* = 0.88 and *p* = 0.69).

**EMG with Dimitrov Index (DI)**

We assessed whether DI differed between the ME/CFS-on and ME/CFS-off with a two-way ANOVA with Groups and Blocks as factors as done in the main document.

For the slopes within each of the 5 blocks, we found no significant main effects of Groups or significant Groups x Blocks interaction (Groups main effect: F(1,15) = 2.64, *p* = 0.12; Interaction: F(4,60) = 0.77, *p* = 0.54). We also directly compared the ME/CFS-on and ME/CFS-off at each of the 5 blocks with independent *t*-tests: there was no significant group difference (*p* = 0.73, 0.39, 0.19, 0.73, 0.73 across the five blocks).

For the DI average at each of the 5 blocks, we found no significant main effects of Groups or significant Groups x Blocks interaction (Groups main effect: F(1,15) = 1.83, *p* = 0.20; Interaction: F(4,60) = 1.47, *p* = 0.22). We also directly compared the ME/CFS-on and ME/CFS-off at each of the 5 blocks with independent *t*-tests: there was no significant group difference (*p* = 0.79, 0.79, 0.79, 0.79, 0.76 across the five blocks).

**EEG in the C3 channel**

We assessed whether EEG power differed between the ME/CFS-on and ME/CFS-off in the 4 frequency bands (theta, mu, beta, gamma) with a two-way ANOVA with Groups and Blocks as factors as done in the main document.

For the slopes within each of the 5 blocks, we found no significant main effects of Groups or significant Groups x Blocks interaction for any of the 4 frequency bands (Groups main effect: F(1,15) = 1.80, 0.91, 0.22, 2.15, *p* = 0.19, 0.35, 0.64, and 0.18 for the theta, mu, beta, gamma, respectively; Interaction: F(4,60) = 0.20, 0.85, 0.87, 0.08, *p* = 0.93, 0.49, 0.48, and 0.98).

We also directly compared the ME/CFS-on and ME/CFS-off at each of the 5 blocks with independent *t*-tests:

- In the theta band, there was no significant difference between the ME/CFS-on and ME/CFS-off (*p* = 0.93, 0.87, 0.87, 0.87, 0.87 across the five blocks).
- In the mu band, there was no significant difference between the ME/CFS-on and ME/CFS-off (*p* = 0.87, 0.87, 0.87, 0.93, 0.87 across the five blocks).
- In the beta band, there was no significant difference between the ME/CFS-on and ME/CFS-off (*p* = 0.87, 0.87, 0.87, 0.87, 0.87 across the five blocks).
- In the gamma band, there was no significant difference between the ME/CFS-on and ME/CFS-off (*p* = 0.87, 0.87, 0.87, 0.30, 0.87 across the five blocks).

For the average at each of the 5 blocks, we found no significant main effects of Groups or significant Groups x Blocks interaction for any of the 4 frequency bands (Groups main effect: F(1,15) = 2.67, 3.88, 3.01, 3.21, *p* = 0.12, 0.07, 0.10, and 0.095 for the theta, mu, beta, gamma, respectively; Interaction: F(4,60) = 0.50, 0.81, 0.93, 1.09, *p* = 0.73, 0.52, 0.44, and 0.36).

We also directly compared the ME/CFS-on and ME/CFS-off at each of the 5 blocks with independent *t*-tests:

- In the theta band, there was no significant difference between the ME/CFS-on and ME/CFS-off (*p* = 0.31, 0.31, 0.31, 0.31, 0.31 across the five blocks).
- In the mu band, there was no significant difference between the ME/CFS-on and ME/CFS-off (*p* = 0.31, 0.31, 0.31, 0.31, 0.31 across the five blocks).
- In the beta band, there was no significant difference between the ME/CFS-on and ME/CFS-off (*p* = 0.31, 0.31, 0.31, 0.41, 0.31 across the five blocks).
- In the gamma band, there was no significant difference between the ME/CFS-on and ME/CFS-off (*p* = 0.31, 0.41, 0.31, 0.31, 0.31 across the five blocks).

**BOLD**

Whole brain

We assessed whether BOLD differed between the ME/CFS-on and ME/CFS-off with a two-way ANOVA with Groups (ME/CFS-on and ME/CFS-off ) and the five Blocks with 3dLME as in the main document.

Groups main effect: There were no clusters surviving the statistical threshold.

Groups x Blocks interaction: There was a small cluster in the anterior cingulate (ACC) with 157 voxels. We report this result even if it does not meet the statistical threshold for cluster size which was set at 201 (see *2.5.2 MRI* in the main document). The interaction was caused by the opposite direction of BOLD across the five blocks, with the ME/CFS-off decreasing and the ME/CFS-on remaining stable. We measured the slopes across the five blocks for each group: ME/CFS-off had a slope = -0.19, *p* = 0.10 and ME/CFS-on had a slope = 0.04, *p* = 0.54, which gave a significant difference (*p* = 0.04). The contrast at each block did not reveal any group difference (all *p* > 0.18).

ROI analysis

We assessed whether BOLD differed between the ME/CFS-on and ME/CFS-off in the same ROI as in the main document (Fig. 6A-B) that included the bilateral primary motor cortex, pre-motor cortex, supplementary motor area, and somatosensory cortex (M1, PM, SMA, SS, respectively). Here we ran two-way ANOVA with Groups and Blocks as factors as done in the main document.

There was no cluster with a significant Groups main effect. There was only 1 cluster with a significant Groups x Blocks interaction. The cluster with a significant Groups x Blocks interaction was the left M1 (F(4, 60) = 2.27, *p* = 0.054). Post hoc *t*-test before FDR correction revealed the ME/CFS-on had significantly higher BOLD than the ME/CFS-off at the F_n+2_ block (*p* = 0.049), but after FDR correction there were no significant group difference at any blocks (all *p* > 0.24).
